# The association between insomnia (related symptoms) and glycaemic control: a systematic review and meta-analysis

**DOI:** 10.7189/jogh.15.04016

**Published:** 2025-02-07

**Authors:** Yiming Chen, Enyu Tong, Yufeng Rao, Evan YW Yu, Maurice Zeegers, Anke Wesselius

**Affiliations:** 1 School of Nutrition and Translational Research in Metabolism, Maastricht University, Maastricht, Limburg, the Netherlands; 2 Department of Epidemiology, Maastricht University, Maastricht, Limburg, the Netherlands; 3 Department of Health, Ethics and Society, Maastricht University, Maastricht, Limburg, the Netherlands; 4 Key Laboratory of Environmental Medicine and Engineering of Ministry of Education, and Department of Epidemiology & Biostatistics, School of Public Health, Southeast University, Nanjing, Jiangsu, China

## Abstract

**Background:**

Insomnia characterised by difficulties in falling asleep and maintaining sleep, and early awaking, is a prevalent worldwide sleep disorder. While previous studies have suggested an association between insomnia and adverse glycaemic control, the evidence remains inconclusive. Therefore, this meta-analysis aims to explore this association.

**Methods:**

Insomnia was assessed based on defined criteria, including related symptoms such as poor sleep quality and low sleep efficiency. Glycaemic control was evaluated using indicators such as fasting plasma glucose, haemoglobin A1c, and the presence of diabetes. A literature search was performed in PubMed, Web of Science, and Scopus. The quality of the included studied was assessed using The Newcastle-Ottawa Scale. Effect sizes, including odds ratio, relative risk, mean difference, and standard mean difference, were chosen based on data type. Forest plots visually displayed pooled effect sizes and corresponding 95% confidence intervals, while the *I*^2^ test calculated heterogeneity. Meta-regression and subgroup analysis explored potential sources of heterogeneity. Leave-one-out sensitivity analysis assessed result robustness, and Begg’s and Egger’s tests evaluated publication bias.

**Results:**

Ninety-one articles, comprising 84 are cross-sectional studies, (five are case-control studies, and two are cohort studies) with 2 217 521 participants, were included. Ten separate meta-analyses were conducted based on variable type (binary/continuous), study design (cross-sectional, case-control, or cohort), and measurement of exposures/outcomes. All meta-analyses indicated a positive association between insomnia (related symptoms) and adverse glycaemic control. However, three meta-analyses showed significant heterogeneity, and three lacked robustness. No publication bias was detected across any of the analyses.

**Conclusions:**

Insomnia is likely associated with adverse glycaemic control. As the included studies are observational, future research should prioritise diverse methodologies and robust study designs to further explore this complex relationship.

**Keywords:**

insomnia, insomnia related symptoms, glycaemic control, systematic review, meta-analysis

**Registration:**

PROSPERO CRD42024491688.

According to the estimation from The International Diabetes Federation [1], the global incidence of diabetes among adults 20–79 was 537 million, accounting for approximately one-tenth of the world population. Projections indicate a steady increase, reaching 643 million by 2030 and 783 million by 2045. Concurrently, the direct health expenditure on diabetes worldwide was 966 billion USD in 2021, with forecasts predicting an escalation to 1.03 trillion USD by 2030 and 1.05 trillion USD by 2045. This alarming escalation underscores the urgent need for effective strategies to manage and mitigate the impact of diabetes on global health systems.

Given the impact of glucose levels on diabetes, certain parameters, such as fasting plasma glucose (FPG) and haemoglobin A1c (HbA1c), are considered as risk factors and are instrumental in identifying potential diabetic individuals [2–4]. Adverse glycaemic control in patients with diabetes is closely related to an increased risk of chronic complications, diabetes-related endpoints, myocardial infarction, and all-cause mortality [5]. Consequently, regardless of diabetes status, maintaining optimal glucose levels is imperative to reduce the risk of debilitating health outcomes.

Insomnia, a prevalent sleep disorder, characterised by difficulty in falling asleep, difficulty in maintaining sleep, and early awaking, can disrupt both cognitive and physical functions in affected individuals [6,7]. Despite its widespread impact, the association between insomnia and glycaemic control has received limited attention.

In the pathophysiology of insomnia, hyperarousal during sleep and wakefulness plays a central role. This includes increased metabolic rate throughout sleep and wakefulness, elevated levels of cortisol and adrenocorticotropic hormone during the early sleep period, reduced parasympathetic tone affecting heart rate variability, and heightened high-frequency electroencephalographic activity during non-rapid eye movement sleep [8].

With roughly one-third of the global population affected by insomnia, understanding its potential implications for glycaemic control is crucial. Laboratory evidence has suggested that insomnia might induce excessive activation of the autonomic sympathetic nervous system and consequently cause insulin resistance and impaired glucose tolerance [9,10]. However, previous observational studies examining the relationship between insomnia and glycaemic control have yielded mixed results. A cross-sectional study in Japan found that insomnia was significantly associated with higher HbA1c levels in Japanese men after adjusting for potential confounders [11]. Similarly, O et al. [12] reported that older adults with type 2 diabetes (T2D) and insomnia had higher HbA1c levels and a greater proportion of adverse glycaemic control (HbA1c ≥7%) compared to those without insomnia. Comparable findings were observed in two additional studies [13,14]. However, not all studies support this association. A cross-sectional study by Yoshida et al. [15] revealed no significant difference in HbA1c levels between participants with and without insomnia, and another study choosing FPG as the indicator for glucose levels also reported no association [16]. In a study including a total of 755 males under 65 years old, no significant difference in occurrence of insomnia between groups of adverse/normal glycaemic control was found [17]. Considering the inconsistent results of previous studies, it is necessary to ascertain the association between insomnia and glycaemic control.

To address this gap in understanding, a comprehensive meta-analysis was conducted to systematically explore the association between insomnia and glycaemic control. The research protocol for this meta-analysis has been registered with PROSPERO under the identifier: CRD42024491688. Through synthesising existing evidence, this study aims to provide clarity on the potential impact of insomnia on glycaemic control, contributing to a better understanding of the interplay between sleep disorders and metabolic health.

## METHODS

This review follows the PRISMA 2020 reporting guidelines [18].

### Search strategy

A comprehensive literature search was performed in PubMed, Web of Science, and Scopus, which used the following medical subject heading terms and Boolean operators: (‘sleep initiation and maintenance disorders’ OR ‘disorders of initiating and maintaining sleep’ OR ‘DIMS’ OR ‘sleep initiation dysfunction*’ OR ‘sleep initiation’ OR ‘initiating sleep’ OR ‘early awakening’ OR ‘wake up early’ OR ‘waking up early’ OR ‘wakeful*’ OR ‘sleeplessness’ OR ‘sleepless’ OR ‘sleep difficult*’ OR ‘difficulty in falling asleep’ OR ‘’fall asleep OR falling asleep’ OR ‘difficulty in maintaining sleep’ OR ‘maintain sleep’ OR ‘sleep maintenance’ OR ‘maintaining sleep’ OR ‘sleep impairment’ OR ‘sleep disturbance’ OR ‘sleep disorder’ OR ‘insomnia’ OR ‘sleep quality’ OR ‘poor sleep’ OR ‘sleep efficiency’) AND (‘glycaemic control’ OR ‘blood glucose’ OR ‘glycated haemoglobin’ OR ‘glycated serum proteins’ OR ‘glycosuria’ OR ‘blood glucose control’ OR ‘fasting plasma glucose’ OR ‘fasting blood glucose’ OR ‘HbA1c’ OR ‘GSP’ OR ‘urine glucose’ OR ‘postprandial plasma glucose’). The search was limited to articles published up to 2 May 2024, the date closely before the submitting date of the article (Table S1 in the **Online Supplementary Document**). Furthermore, articles that met the inclusion criteria but were not initially identified through the search strategy were incorporated if discovered in the references of previous articles.

### Inclusion and exclusion criteria

#### Inclusion criteria

To ensure relevance and applicability, this meta-analysis included only observational study designs – namely cohort, case-control, and cross-sectional studies. This choice was made due to the ethical and practical difficulties associated with using insomnia as an intervention in experimental studies. Studies were eligible if they involved any demographic group and provided data on insomnia or related symptoms alongside measures of glycaemic control. The studies needed to clearly define insomnia and related symptoms, such as poor sleep quality and low sleep efficiency. Additionally, eligible studies had to report on indicators of glycaemic control, including HbA1c, FPG, urine glucose, glycated serum proteins, and postprandial plasma glucose. Diabetes was also considered an outcome due to its known impact on glycaemic control.

#### Exclusion criteria

Articles were excluded if they did not provide primary data, such as theoretical models, case reports, protocols, conference abstracts, editorials, comments, and review articles. Studies with incomplete or invalid data were also excluded to maintain the quality and accuracy of the analysis. Duplicate publications or articles with overlapping data were removed to avoid redundancy. Experimental laboratory studies involving either human or non-human subjects were excluded, as they did not align with the observational study design required for this meta-analysis. Finally, articles that were not accessible in full text were excluded to ensure a thorough evaluation of the included studies.

### Study selection and data extraction

Two independent reviewers conducted a dual-stage screening of articles, initially evaluating titles and abstracts, followed by a comprehensive assessment of full texts.

Subsequently, both reviewers autonomously extracted crucial information from the selected articles. These were: Study data, including descriptive information such as the total number of participants, how they were categorised based on exposure and/or outcome, and the mean ± standard deviation of exposure or outcome measurements for each category. Other extracted information included study design, primary author, publication year, geographic region, age, gender, and diabetes status.

After the independent study selection, two reviewers cross-checked their results and discussed any inconsistencies to reach a consensus on the final selected articles. A similar procedure was followed after data extraction to ensure accuracy. In cases where disagreements persisted, the issue was resolved through a collaborative panel discussion involving all reviewers.

### Study risk of bias assessment

The Newcastle-Ottawa Scale (NOS) [19] was used for evaluating the risk of bias of cohort studies and case-control studies, and an adapted version of NOS was used for cross-sectional studies [20] (Appendix S1 in the **Online Supplementary Document**). The NOS, whose scores range from zero to nine for cohort studies and case-control studies, and range from zero to seven for cross-sectional studies, includes three domains to assess bias in selection, comparability, and exposure/outcome. For a study, a NOS score being six or higher can be considered low risk of bias; correspondingly, a score ranging from four to five is considered moderate risk of bias, and a score lower than four is considered high risk of bias. Two independent reviewers conducted the assessment.

Following the independent risk of bias assessments, two reviewers reviewed and discussed their results to resolve any inconsistencies in the NOS scores assigned to each study. If disagreements persisted, a resolution was achieved through a collaborative panel discussion involving all reviewers.

### Statistical analysis

Review Manager 5.3 (The Cochrane Collaboration) was selected for its advanced capabilities in conducting meta-analyses and generating detailed forest plots, while Stata 12.0 (StataCorp LLC, College Station TX, USA) was chosen for its robust features in creating comprehensive figures for sensitivity analysis and publication bias assessments. The combination of these software programmes facilitated thorough and precise statistical analysis. Based on data type of exposure/outcome (binary or continuous variables), study design (case-control, cohort, or cross-sectional), and the measurement indicator for exposure/outcome, the odds ratio (OR), relative risk (RR), mean difference (MD), and standard mean difference (SMD) were chosen as the effect sizes correspondingly. In detail, for meta-analyses including cross-sectional studies and case-control studies with binary exposures and outcomes, OR was chosen; for meta-analyses including cohort studies with binary exposures and outcomes, RR was chosen; for meta-analyses whose exposures or outcomes were same continuous measurement indicators and outcomes or exposures were binary variables, MD was chosen; for meta-analyses whose exposures or outcomes were different continuous measurement indicators and outcomes or exposures were binary variables, SMD was chosen. The random-effects model was chosen for all meta-analyses. Forest plots were used for demonstrating pooled effect sizes and corresponding 95% confidence intervals (CIs) visually. Heterogeneity was assessed using the *I*^2^ test, with *I*^2^>50% indicating substantial heterogeneity. Meta-regression and subgroup analysis were employed to explore sources of heterogeneity. Specifically, subgroup analysis was conducted if fewer than 10 studies were included in a meta-analysis; otherwise, meta-regression was prioritised, followed by subgroup analysis if *P* < 0.10. A leave-one-out sensitivity analysis was performed to assess the robustness of the pooled results. For evaluating publication bias, the Begg’s test (presented as funnel plots) and Egger’s test were performed, and if *P* < 0.05, it is considered publication bias.

## RESULTS

### Literature search

#### Article selection overview

**Figure 1:**
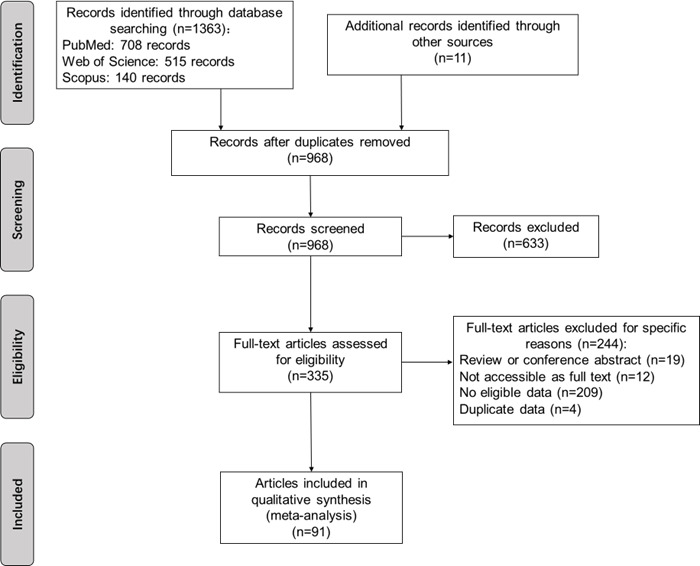
The article selection process.

Following the comprehensive search, a total of 1374 articles were initially identified. Initially, 406 duplicates were removed. Subsequently, 633 articles were excluded based on evaluations of titles and abstracts. Further, 244 articles were excluded for various specific reasons during full-text reading. Finally, a final selection of 91 articles [11–17,21–104] was included in the meta-analysis, encompassing a total of 2 217 521 participants (**Figure 1**).

#### Study design distribution

Among the incorporated articles, 84 were cross-sectional studies [11–17,21,22,25–27,29–51,53–66,68–94,96–102,104], five were case-control studies [23,24,28,52,95], and two were cohort studies [67,103] (Table S2 in the **Online Supplementary Document**).

#### Risk of bias

Among the included studies, 41 (48.81%) cross-sectional studies, five (100%) case-control studies, and two (100%) cohort studies were appraised as having a low risk of bias [14,21–26,28,29,31–34,37–40,47,49,51–54,56,57,59,61,63,65–73,77,78,81,82,84,88,95,97,100,102,103]. In contrast, among the remaining cross-sectional studies, 41 (48.81%) were deemed to have moderate risk of bias [11–13,15–17,27,35,36,41–46,48,50,55,58,60,62,64,74–76,79,80,85–87,89–94,96,98,99,101,104], while two (2.38%) were classified as having high risk of bias [30,83]. Notably, most studies categorised as moderate or high risk of bias exhibited lower scores particularly in the dimensions of Selection and Comparability. Studies often faced Selection bias due to non-random participant sampling, leading to potential non-representativeness. In terms of Comparability, many studies were cross-sectional with poorly matched groups, limiting their ability to establish causal relationships and increasing the risk of confounding (Table S3 in the **Online Supplementary Document**).

### Insomnia (related symptoms) and glycaemic control

Given the limited inclusion of only five case-control studies [23,24,28,52,95], we decided to combine the case-control data with cross-sectional data into the same analyses. Despite the design differences between case-control and cross-sectional studies, the two types of studies often use the same statistical models to combine the data and estimate effect sizes, and thereby effect sizes are often the same (like OR, MD, and SMD). Thus, it is statistically reasonable to combine case-control and cross-sectional studies. To make sure we covered all the different types of data, we carefully sorted them based on whether they were binary or continuous variables, the study design (cross-sectional/case-control, or cohort), and how exposures/outcomes were measured. This detailed approach led us to conduct 10 separate meta-analyses, each customised to fit the characteristics of the studies we included (**Table 1**). We summarised the results of these analyses visually in Figures S1–10 in the **Online Supplementary Document**, using forest plots. These plots give a clear picture of the various effects we observed across different exposures and outcomes, helping to understand the findings better.

**Table 1:** The results of meta-analyses

Number of articles	Exposure/outcome	Sample size	Effect size (95% CI), *P-*value	Heterogeneity (*I*^2^)	*P-*value for publication bias
Cross-sectional studies and case-control studies: binary exposure and binary outcome					
21 [27,30,33,41–43,48,61,63,64,68,69,72,79,82,84,86-88,94,100]	Sleep quality (high/low) and glycaemic control (adverse/normal)	47 009	OR = 1.63 (1.30, 2.04), *P* < 0.001	88.4%	0.606
6 [11,14,17,36,62,89]	Insomnia (yes/no) and glycaemic control (adverse/normal)	9911	OR = 1.87 (1.19, 2.93), *P* = 0.006	78.0%	0.319
7 [37,46,70,72,76,83,95]	Sleep quality (high/low) and diabetes (yes/no)	22 202	OR = 2.28 (1.84, 2.82), *P* < 0.001	32.2%	0.819
4 [45,52,58,97]	Insomnia (yes/no) and diabetes (yes/no)	31 152	OR = 1.32 (1.12, 1.56), *P* = 0.001	35.9%	0.399
Cross-sectional studies and case-control studies: binary exposure and continuous outcome					
23 [21,24–26,29,31,39,40,49,51,53,54,73-75,79,80,85,91,92,96,101,102]	Sleep quality (high/low) and glucose levels	6072	SMD = 0.14 (0.06, 0.22), *P* < 0.001	38.6%	0.056
7 [12,13,15,16,57,60,67,98,105]	Insomnia (yes/no) and glucose levels	6745	SMD = 0.16 (0.07, 0.24), *P* < 0.001	14.6%	0.969
Cross-sectional studies and case-control studies: continuous exposure and binary outcome					
15 [22,23,28,32,34,38,47,50,59,65,77,78,95,99,104]	Sleep quality scores and diabetes (yes/no)	7939	SMD = 0.81 (0.40, 1.21), *P* < 0.001	97.6%	0.353
2 [35,55]	Sleep quality scores and glycaemic control (adverse/normal)	1431	MD = 0.65 (0.10, 1.20), *P* = 0.020	<0.1%	NA
6 [44,56,66,81,90,93]	Sleep efficiency and diabetes (yes/no)	498	MD = −2.73 (−4.31, −1.16), *P* < 0.001	18.1%	0.971
Cohort studies: binary exposure and binary outcome					
2 [67,103]	Insomnia or related symptoms (yes/no) and diabetes (yes/no)	84 562	RR = 1.07 (1.04, 1.11), *P* < 0.001	<0.1%	NA

CI – confidence interval, MD – mean difference, NA – not applicable, OR – odds ratio, RR – relative risk, SMD – standard mean difference.

#### Cross-sectional studies and case-control studies with both binary exposures and outcomes

Four meta-analyses were performed (Figures S1–4 in the **Online Supplementary Document**).

In the meta-analysis comparing the occurrence of low sleep quality between groups of adverse/normal glycaemic control, 21 studies [27,30,33,41–43,61,63,64,68,69,72,79,82,84,86–88,94,100] with a total number of 47 009 participants were included. The result showed that the occurrence of low sleep quality in participants with adverse glycaemic control was higher than that with normal glycaemic control (OR = 1.63; 95% CI = 1.30, 2.04, *P* < 0.001). The *I*^2^ test indicated heterogeneity among the studies (*I*^2^ = 88.4%).

In the meta-analysis comparing the occurrence of insomnia between groups of adverse/normal glycaemic control, six articles [11,14,17,36,62,89] with a total number of 9911 participants were included. The synthesised findings revealed a positive association, with individuals exhibiting adverse glycaemic control being 1.87 times more likely to experience insomnia compared to those with normal glycaemic control (OR = 1.87; 95% CI = 1.19, 2.93, *P* = 0.006). The *I*^2^ test indicated heterogeneity among the studies (*I*^2^ = 78.0%).

In the meta-analysis comparing the occurrence of low sleep quality between groups with/without diabetes, seven articles [37,46,70,72,76,83,95] with a total number of 22 202 participants were included. The synthesised findings revealed a positive association, indicating that individuals with low sleep quality were 2.28 times more likely to have diabetes compared to those without low sleep quality (OR = 2.28; 95% CI = 1.84, 2.82, *P* < 0.001). The *I*^2^ test did not indicate heterogeneity among the studies (*I*^2^ = 32.2%).

In the meta-analysis comparing the occurrence of insomnia between groups with/without diabetes, four [45,52,58,97] articles with a total number of 31 152 participants were included. The synthesised findings revealed an association, indicating that individuals with insomnia were 1.32 times more likely to develop diabetes compared to those without insomnia (OR = 1.32; 95% CI = 1.12, 1.56, *P* = 0.001). The *I*^2^ test did not indicate heterogeneity among the studies (*I*^2^ = 35.9%).

#### Cross-sectional studies and case-control studies with binary exposures and continuous outcomes

Two meta-analyses were performed (Figures S5–6 in the **Online Supplementary Document**).

In the meta-analysis comparing the glucose levels of groups with high/low sleep quality, 23 articles [21,24–26,29,31,39,40,49,51,53,54,73–75,79,80,85,91,92,96,101,102] with a total number of 6072 participants were included. In consideration of the different measurement indicators for glucose levels, SMD was chosen as the effect size. The synthesised result indicated that the glucose levels of group with low sleep quality were higher than that those with low sleep quality (SMD = 0.14; 95% CI = 0.06, 0.22, *P* < 0.001). The *I*^2^ test did not indicate heterogeneity among the studies (*I*^2^ = 38.6%).

In the meta-analysis comparing the glucose levels of groups with/without insomnia, seven articles [12,13,15,16,57,60,98] with a total number of 6745 participants were included. The synthesised result revealed a distinction. Specifically, individuals with insomnia exhibited higher glucose levels compared to those without insomnia (SMD = 0.16; 95% CI = 0.07, 0.24, *P* < 0.001). The *I*^2^ test did not indicate heterogeneity among the studies (*I*^2^ = 14.6%)

#### Cross-sectional studies and case-control studies with continuous exposures and binary outcomes

Three meta-analyses were performed (Figures S7–9 in the **Online Supplementary Document**).

In the meta-analysis comparing the sleep quality scores between groups with/without diabetes, 15 articles [22,23,28,32,34,38,47,50,59,65,77,78,95,99,104] with a total number of 7939 participants were included. In consideration of the different measurement indicators for sleep quality, SMD was chosen as the effect size. The synthesised result indicated that the sleep quality of the group with diabetes were lower than that without diabetes (SMD = 0.81; 95% CI = 0.40, 1.21, *P* < 0.001). The *I*^2^ test indicated heterogeneity among the studies (*I*^2^ = 97.6%).

In the meta-analysis comparing the sleep quality scores between groups of adverse/normal glycaemic control, two articles (both used Pittsburgh Sleep Quality Index as the indicator for exposure) with a total number of 1431 [35,55] participants were included. The synthesised result indicated that individuals with adverse glycaemic control exhibited lower sleep quality scores compared to those with normal glycaemic control (MD = 0.65; 95% CI = 0.10, 1.20, *P* = 0.020). The *I*^2^ test did not indicate heterogeneity among the studies (*I*^2 ^< 0.1%).

In the meta-analysis comparing the sleep efficiency between groups with/without diabetes, six articles [44,56,66,81,90,93] with a total number of 498 participants were included. The synthesised result indicated individuals with diabetes experienced a lower sleep efficiency compared to those without diabetes (MD = −2.73; 95% CI = −4.31, −1.16, *P* < 0.001). The *I*^2^ test did not indicate heterogeneity among the studies (*I*^2^ = 18.1%).

#### Cohort studies with both binary exposures and outcomes

One meta-analysis comparing the risk of diabetes between groups with/without insomnia (related symptoms) was performed (Figure S10 in the **Online Supplementary Document**). In this meta-analysis, two articles [67,103] with a total number of 84 562 participants were included. The synthesised result indicated that the risk of diabetes in group with insomnia (related symptoms) was higher than that without insomnia (related symptoms) (RR = 1.07; 95% CI = 1.04, 1.11, *P* < 0.001). The *I*^2^ test did not indicate heterogeneity among the studies (*I*^2 ^< 0.1%).

### Meta-regressions and subgroup analyses

To identify potential sources of heterogeneity, meta-regressions and subgroup analyses were conducted based on the following criteria:

a) publication year

b) measurement scale for exposures/outcomes

c) geographic region

d) mean/median age

e) male proportion

f) diabetes status.

Among the three meta-analyses exhibiting heterogeneity, two meta-analyses included more than 10 studies:

a) the occurrence of low sleep quality between groups of adverse/normal glycaemic control

b) the sleep quality scores between groups with/without diabetes.

Thus, meta-regression and subgroup analysis were considered to perform. However, the sources of heterogeneity remained unidentified after mete-regression and subgroup analysis. In the meta-analysis examining the occurrence of insomnia among those with adverse vs normal glycaemic control, only six studies were included, which was not applicable for meta-regression, and thus subgroup analysis was performed directly. After dividing the included articles into subgroups based on whether all participants had T2D, the variation in outcomes between the groups highly decreased (**Table 2**).

**Table 2:** The subgroup analysis for the meta-analysis comparing the occurrence of insomnia between groups of adverse/normal glycaemic control

Subgroup	Number of articles	OR (95% CI), *P-*value	Heterogeneity (*I*^2^)
All participants suffered from T2D	1 [14]	5.67 (3.03, 10.60), *P* < 0.001	NA
Not all participants suffered from T2D	5 [11,17,36,62,89]	1.37 (1.15, 1.63), *P* < 0.001	2.0%
Total	6 [11,14,17,36,62,89]	1.87 (1.19, 2.93), *P* = 0.006	78.0%

CI – confidence interval, NA – not applicable, OR – odds ratio, T2D – type 2 diabetes.

### Sensitivity analyses and publication bias

The leave-one-out sensitivity analysis showed similar results, indicating results are totally robust and reliable. Only three meta-analyses including few studies did not show the robust results (Figure S11 in the **Online Supplementary Document**):

a) the meta-analysis comparing the occurrence of insomnia between groups with/without diabetes (when leaving out the study by Henriksen et al. [52])

b) the meta-analysis comparing the sleep quality scores between groups of adverse/normal glycaemic control (when leaving out the study by Hung et al. [55])

c) the meta-analysis comparing the risk of diabetes between groups with/without insomnia (related symptoms) (when leaving out the study by LeBlanc et al. [67]).

There was no publication bias in all meta-analyses (**Table 1**), and the funnel plots are presented in Figure S12 in the **Online Supplementary Document**.

## DISCUSSION

This study conducted 10 distinct meta-analyses to investigate the relationship between insomnia and glycaemic control. The findings suggest that insomnia symptoms are likely associated with an increased risk of adverse glycaemic control.

This result aligns with a meta-analysis whose literature search was performed in 2018, demonstrating that in individuals with T2D, insomnia (symptoms) was associated with higher HbA1c levels (MD = 0.23%, 95% CI = 0.1, 0.4) and higher fasting glucose levels (MD = 0.40 mmol/L, 95% CI = 0.2, 0.7) [106]. Previous studies have tried to explain the potential mechanisms through which insomnia contributes to adverse glycaemic control. Insomnia may induce excessive activation of the autonomic sympathetic nervous system, resulting in insulin resistance and impaired glucose tolerance [9,10]. Moreover, insomnia probably influences the activity of hypothalamic-pituitary-adrenal axis, which leads to evening elevations of cortisol, and thereby affects insulin signalling and reduces insulin secretion [107]. Furthermore, the reduction in slow-wave sleep caused by insomnia can lead to a reduction in insulin sensitivity, which is harmful to the dynamic balance of systemic glucose homeostasis [108,109]. Additionally, insomnia has been linked to heightened systemic inflammation, further exacerbating insulin resistance [110,111]. Finally, individuals with insomnia may have abnormal levels hormones such as leptin and ghrelin, which regulate satiety and hunger respectively, thereby disrupting energy balance and impacting glucose metabolism and homeostasis [9,112].

Given the association between insomnia and adverse glycaemic control, it is crucial to implement measures to address insomnia effectively. Previous studies suggest, the primary treatments can generally be categorised in cognitive behavioural therapy for insomnia and pharmacological treatments. Cognitive behavioural therapy for insomnia, which is considered as the gold standard in treating insomnia, consists of the following major components: stimulus control, sleep restriction, relaxation techniques, cognitive therapy, and sleep hygiene education [113,114]. As for pharmacological treatments, benzodiazepines, benzodiazepine-receptor agonists, and melatonin have been commonly used for treating insomnia [113,114]. However, the long-term use of benzodiazepines and benzodiazepine-receptor agonists carries risks of tolerance and dependency, which limits their recommended use [113,114]. For melatonin, excessive intake may lead to side effects such as amnesia or a ‘melatonin hangover,’ characterised by increased difficulty falling asleep, or waking up after a few hours and struggling to return to sleep [115].

This study has some limitations. First, given all the included studies were observational, with a significant proportion being cross-sectional, the predominance of cross-sectional studies may affect the overall level of evidence, particularly as causation cannot be established and there is a possibility of reverse causation. Second, three meta-analyses with small numbers of studies produced non-robust results. This is likely due to the limited sample sizes and the influence of individual studies with disproportionately large sample sizes [52,55,67]. This variability affects the overall reliability of the findings and highlights the need for larger, more consistent studies to achieve more robust conclusions. Third, since the design differences (such as temporal dimension, sample selection, and group comparability) between the case-control studies and cross-sectional studies, the combination could potentially increase the heterogeneity and the bias of the merged results. The combined results should be interpreted with caution. In this study, heterogeneity was observed in three out of ten meta-analyses. Specifically, the meta-analysis examining the occurrence of insomnia among groups with adverse vs normal glycaemic control revealed a potential source of heterogeneity based on whether participants suffered from T2D. Notably, subgroup analysis indicated a higher likelihood of adverse glycaemic control among participants with T2D compared with those without, suggesting a possible association between T2D and insomnia-induced adverse glycaemic control.

However, in the remaining two meta-analyses with heterogeneity, the sources of variation remained elusive even after meta-regression and subgroup analysis. This was possible partly because the combination of different study designs into the same models could potentially increase the heterogeneity of the merged results. Meanwhile, the challenge may also stem from the substantial number of articles included in these analyses (20 and 23 articles, respectively), posing difficulties in adequately controlling for various confounding factors. Among these confounders, comorbid sleep disorders such as habitual snoring, obstructive sleep apnoea, and restless leg syndrome, which have been linked to adverse glycaemic control in previous research, were frequently unaccounted for in the included studies [116–123]. Similarly, mental health disorders like depression and anxiety, which often coexist with insomnia, could also influence glycaemic regulation [7,124-126]. Furthermore, the use of medication, particularly those targeting glycaemic control, may introduce additional variability in our findings. Moreover, the included studies often differed in indicators for exposures (related symptoms) and glycaemic control. Even in the same indicator, the standards were also sometimes different. Taking HbA1c as an example, many included studies chose HbA1c as the indicator for glucose levels. Among them, some used HbA1c ≥6.5% [27,72] as the standard for adverse glycaemic control, while some others used HbA1c >6.5% [48,63], HbA1c ≥7% [30,33,41,43], or HbA1c >7% [33,41,87] as the standards. Different indicators and corresponding standards could also cause the heterogeneity. Finally, the participants from all over the world had diverse demographic backgrounds, and many important demographic factors were not always mentioned and thereby were difficult to correct.

While this study possesses certain limitations, its findings remain valuable for several reasons. First, it is important to acknowledge that most of the studies included in this analysis are cross-sectional, which may affect the overall level of evidence. However, despite this inherent limitation, observational studies provide valuable insights into real-world scenarios and can offer crucial evidence to inform clinical decision-making and further research directions.

Second, three of these analyses yielded non-robust results. Nevertheless, it is essential to contextualise these findings within the broader scope of the study. Given that these three meta-analyses were based on a relatively small number of articles (four, two, and two, correspondingly), such outcomes are not uncommon in meta-analytical research and do not necessarily undermine the overall validity of the study's conclusions. Thus, despite these limitations, the study still contributes valuable insights into the relationship between insomnia and glycaemic control, shedding light on an important and clinically relevant topic. Additionally, it is noted that three out of the ten meta-analyses revealed heterogeneity, and efforts to mitigate this heterogeneity were unsuccessful in two of the three. Considering the large total sample sizes of these two meta-analyses, their results were still generally valuable.

## CONCLUSIONS

This study underscores the association between insomnia and adverse glycaemic control. While the findings offer valuable insights, it's essential to acknowledge that this meta-analysis has some limitations. Future research endeavours should focus on the following aspects to minimise the limitations. First, given most of the included studies were cross-sectional, more robust studies with higher levels of evidence should be performed to strengthen the evidence base and elucidate causation more definitively. Second, the non-robustness of some meta-analyses highlights the need for larger, more consistent studies to achieve more robust conclusions. Finally, future meta-analyses in this domain should conduct comprehensive assessments of potential confounding factors to enhance the precision and reliability of meta-analytical outcomes.

## Additional material


Online Supplementary Document
