# Supplementary material for: The association between insomnia (related symptoms) and glycaemic control: a systematic review and meta-analysis
Source: J Glob Health. 2025 Feb 7;15:04016. doi: 10.7189/jogh.15.04016 (PMC11803432; doi:10.7189/jogh.15.04016)
Supplement: Online Supplementary Document [file jogh-15-04016-s001.pdf]

Supplementary Document

Table S1. The detailed search strategy

Database	Search strategy	Search date	Total number of identified articles
PubMed	((("sleep initiation and maintenance disorders"[Mesh]) OR (disorders of initiating and maintaining sleep[Title/Abstract]) OR (DIMS[Title/Abstract]) OR (sleep initiation dysfunction*[Title/Abstract]) OR (sleep initiation[Title/Abstract]) OR (initiating sleep[Title/Abstract]) OR (early awakening[Title/Abstract]) OR (wake up early[Title/Abstract]) OR (waking up early[Title/Abstract]) OR (wakeful*[Title/Abstract])OR (sleeplessness[Title/Abstract]) OR (sleepless[Title/Abstract]) OR (sleep difficult*[Title/Abstract]) OR (difficulty in falling asleep[Title/Abstract]) OR (fall asleep[Title/Abstract]) OR (falling asleep[Title/Abstract]) OR (difficulty in maintaining sleep[Title/Abstract]) OR (maintain sleep[Title/Abstract]) OR (sleep maintenance[Title/Abstract]) OR (maintaining sleep[Title/Abstract]) OR (sleep impairment[Title/Abstract]) OR (sleep disturbance[Title/Abstract]) OR (sleep disorder[Title/Abstract]) OR (insomnia[Title/Abstract]) OR (sleep quality[Title/Abstract]) OR (poor sleep[Title/Abstract]) OR (sleep efficiency[Title/Abstract]))) AND (("glycemic control"[Mesh]) OR ("blood glucose"[Mesh]) OR ("glycated hemoglobin"[Mesh]) OR ("glycated serum proteins"[Mesh]) OR ("glycosuria"[Mesh]) OR (blood glucose control[Title/Abstract]) OR (fasting plasma glucose[Title/Abstract]) OR (fasting blood glucose[Title/Abstract]) OR (HbA1c[Title/Abstract]) OR (GSP[Title/Abstract]) OR (urine glucose[Title/Abstract]) OR (postprandial plasma glucose[Title/Abstract]))) NOT ((meta-analysis[Title]) OR (review[Title]) OR (systematic review[Title]) OR (Mendelian Randomization[Title]))	May 2, 2024	708
Web of Science	(((TI="sleep initiation and maintenance disorders" OR TI="disorders of initiating and maintaining sleep" OR TI="DIMS" OR TI="sleep initiation dysfunction*" OR TI="sleep initiation" OR TI="initiating sleep" OR TI="early awakening" OR TI="wake up early" OR TI="waking up early" OR TI="wakeful*" OR TI="sleeplessness" OR TI="sleepless" OR TI="sleep difficult*" OR TI="difficulty in falling asleep" OR TI="fall asleep" OR TI="falling asleep" OR TI="difficulty in maintaining sleep" OR TI="maintain sleep" OR TI="sleep maintenance" OR TI="maintaining sleep" OR TI="sleep impairment" OR TI="sleep disturbance" OR TI="sleep disorder" OR TI="insomnia" OR TI="sleep quality" OR TI="poor sleep" OR TI="sleep efficiency") OR (AB="sleep initiation and maintenance disorders" OR AB="disorders of initiating and maintaining sleep" OR AB="DIMS" OR AB="sleep initiation dysfunction*" OR AB="sleep initiation" OR AB="initiating sleep" OR AB="early awakening" OR AB="wake up early" OR AB="waking up early" OR AB="wakeful*" OR AB="sleeplessness" OR AB="sleepless" OR AB="sleep difficult*" OR AB="difficulty in falling asleep" OR AB="fall asleep" OR AB="falling asleep" OR AB="difficulty in maintaining sleep" OR AB="maintain sleep" OR AB="sleep	May 2, 2024	515

Database	Search strategy	Search date	Total number of identified articles
	maintenance" OR AB="maintaining sleep" OR AB="sleep impairment" OR AB="sleep disturbance" OR AB="sleep disorder" OR AB="insomnia" OR AB="sleep quality" OR AB="poor sleep" OR AB="sleep efficiency")) AND ((TI="glycemic control" OR TI="blood glucose" OR TI="glycated hemoglobin" OR TI="glycated serum proteins" OR TI="glycosuria" OR TI="blood glucose control" OR TI="fasting plasma glucose" OR TI="fasting blood glucose" OR TI="HbA1c" OR TI="GSP" OR TI="urine glucose" OR TI="postprandial plasma glucose") OR (AB="glycemic control" OR AB="blood glucose" OR AB="glycated hemoglobin" OR AB="glycated serum proteins" OR AB="glycosuria" OR AB="blood glucose control" OR AB="fasting plasma glucose" OR AB="fasting blood glucose" OR AB="HbA1c" OR AB="GSP" OR AB="urine glucose" OR AB="postprandial plasma glucose"))) NOT (TI="meta-analysis" OR TI="review" OR TI="systematic review" OR TI="Mendelian Randomization")		
Scopus	(INDEXTERMS("sleep initiation maintenance disorders") OR TITLE-ABS("disorders of initiating maintaining sleep") OR TITLE-ABS("DIMS") OR TITLE-ABS("sleep initiation dysfunction*") OR TITLE-ABS("sleep initiation") OR TITLE-ABS("initiating sleep") OR TITLE-ABS("early awakening") OR TITLE-ABS("wake up early") OR TITLE-ABS("waking up early") OR TITLE-ABS("wakeful*") OR TITLE-ABS("sleeplessness") OR TITLE-ABS("sleepless") OR TITLE-ABS("sleep difficult*") OR TITLE-ABS("difficulty in falling asleep") OR TITLE-ABS("fall asleep") OR TITLE-ABS("falling asleep") OR TITLE-ABS("difficulty in maintaining sleep") OR TITLE-ABS("maintain sleep") OR TITLE-ABS("sleep maintenance") OR TITLE-ABS("maintaining sleep") OR TITLE-ABS("sleep impairment") OR TITLE-ABS("sleep disturbance") OR TITLE-ABS("sleep disorder") OR TITLE-ABS("insomnia") OR TITLE-ABS("sleep quality") OR TITLE-ABS("poor sleep") OR TITLE-ABS("sleep efficiency")) AND (INDEXTERMS("glycemic control") OR INDEXTERMS("blood glucose") OR INDEXTERMS("glycated hemoglobin") OR INDEXTERMS("glycated serum proteins") OR INDEXTERMS("glycosuria") OR TITLE-ABS("blood glucose control") OR TITLE-ABS("fasting plasma glucose") OR TITLE-ABS("fasting blood glucose") OR TITLE-ABS("HbA1c") OR TITLE-ABS("GSP") OR TITLE-ABS("urine glucose") OR TITLE-ABS("postprandial plasma glucose")) AND NOT INDEX(medline) AND NOT (TITLE("meta-analysis") OR TITLE("review") OR TITLE("systematic review") OR TITLE("Mendelian Randomization")) AND (LIMIT-TO(SUBJAREA,"MEDI")) AND (LIMIT-TO (DOCTYPE,"ar")) AND (LIMIT-TO(LANGUAGE,"English"))	May 2, 2024	140

Table S2. The details of included articles

First Author	Year	Exposure	Indicator	Outcome	Indicator	Sample size	Details of participants				NOS score
							Region	Age*	Gender (male, %)	Diabetes status	
Cross-sectional studies											
Abdu et al. [1]	2023	Sleep quality	Low quality: PSQI ≥ 5; high quality: PSQI < 5	Glucose levels	HbA1c (%)	269	Qatar	51.0 ± 9.5	63.9	T2D population	7
Adler et al. [2]	2017	Sleep quality	SDSC	T1D	Clinical diagnosis	90	Israel	T1D population: 9.94 ± 1.66; non-diabetes population: 9.07 ± 1.73	48.89	T1D population and non-diabetes population	6
Al-Musharaf et al. [3]	2023	Sleep quality	Low quality: PSQI ≥ 5; high quality: PSQI < 5	Glucose levels	FPG (mg/dL)	487	Saudi Arabia	35.19 ± 12.74	34.1	Non-diabetes population	6
Aribas et al. [4]	2015	Sleep quality	Low quality: PSQI > 5; high quality: PSQI ≤ 5	Glucose levels	FPG (mg/dL)	78	Turkey	Low sleep quality population: 50.6 ± 9.2; high sleep quality population: 47.7 ± 9.4	38.46	T2D population	6
Azuma et al. [5]	2019	Sleep quality	Questionnaire	Glucose levels	Adverse glycaemic control: HbA1c ≥ 6.5%; normal glycaemic control: HbA1c < 6.5%	6,025	Japan	≥ 20	57.43	-	4
Bani-Issa et al. [6]	2018	Sleep quality	Low quality: PSQI ≥ 5.5; high quality: PSQI < 5.5	Glucose levels	HbA1c (%)	268	United Arab Emirates	42.4 ± 12.5	38.06	T2D population	6

First Author	Year	Exposure	Indicator	Outcome	Indicator	Sample size	Details of participants				NOS score
							Region	Age*	Gender (male, %)	Diabetes status	
Barakat et al. [7]	2019	Sleep quality	Low quality: PSQI ≥ 8; high quality: PSQI < 8	Glucose levels	Adverse glycaemic control: HbA1c ≥ 7%; normal glycaemic control: HbA1c < 7%	1,211	Jordan	58.8 ± 9.74	44.6	T2D population	3
Barikani et al. [8]	2019	Sleep quality	Low quality: PSQI ≥ 6; high quality: PSQI < 6	Glucose levels	HbA1c (%)	347	Iran	Low sleep quality population: 55.1 ± 9.04; high sleep quality population: 53.3 ± 8.4	26.1	T2D population	6
Barone et al. [9]	2015	Sleep quality	PSQI	T1D	Clinical diagnosis	27	Brazil	T1D population: 26.3 ± 5.1; non-diabetes population: 28.8 ± 5.3	44.44	T1D population and non-diabetes population	6
Bener et al. [10]	2020	Sleep quality	Low quality: PSQI > 5; high quality: PSQI ≤ 5	Glucose levels	Adverse glycaemic control: HbA1c ≥ 7%; normal glycaemic control: HbA1c < 7%	871	Turkey	-	37.89	T2D population	7
Caruso et al. [11]	2014	Sleep quality	SDSC	T1D	Clinical diagnosis	43	Australia	T1D population: 12.0 ± 2.8; non-diabetes population: 11.2 ± 2.8	56.1	T1D population and non-diabetes population	6

First Author	Year	Exposure	Indicator	Outcome	Indicator	Sample size	Details of participants				NOS score
							Region	Age*	Gender (male, %)	Diabetes status	
Cho et al. [12]	2023	Sleep quality	PSQI	Glucose levels	Adverse glycaemic control: HbA1c ≥ 8%; normal glycaemic control: HbA1c < 8%	96	Korea	32.29 ± 9.89	32.1	T1D population	5
Clark et al. [13]	2014	Insomnia	Karolinska Sleep Questionnaire (KSQ): an average frequency of the insomnia symptoms of "at least some times a week"	Glucose levels	Adverse glycaemic control: HbA1c ≥ 6.5%; normal glycaemic control: HbA1c < 6.5%	1,629	Denmark	54 ± 4	71.98	-	5
Colbay et al. [14]	2015	Sleep quality	Low quality: PSQI > 5; high quality: PSQI ≤ 5	T2D	Clinical diagnosis	97	Turkey	T2D population: 51.4 ± 8.3; non-diabetes population: 50.5 ± 9.8	39.36	T2D population and non-diabetes population	6
Çömlek et al. [15]	2021	Sleep quality	PSQI	T1D	Clinical diagnosis	98	Turkey	T1D population: 14.3 ± 1.7; non-diabetes population: 14.1 ± 1.9	48.98	T1D population and non-diabetes population	6
Corrado et al. [16]	2024	Sleep quality	Low quality: PSQI ≥ 5; high quality: PSQI < 5	Glucose levels	HbA1c (%)	117	Italy	Low sleep quality population: 42.4 ± 14.3; high sleep quality population: 38.7 ± 14.4	50.43	T1D population	6

First Author	Year	Exposure	Indicator	Outcome	Indicator	Sample size	Details of participants				NOS score
							Region	Age*	Gender (male, %)	Diabetes status	
Culver et al. [17]	2020	Sleep quality	Low quality: PSQI > 5; high quality: PSQI ≤ 5	Glucose levels	FPG (mg/dL)	31	USA	Low sleep quality population: 28.8 ± 10.0; high sleep quality population: 29.8 ± 10.3	54.84	Non-diabetes population	6
Cunha et al. [18]	2008	Sleep quality	Low quality: PSQI > 5; high quality: PSQI ≤ 5	Glucose levels	Adverse glycaemic control: HbA1c ≥ 7%; normal glycaemic control: HbA1c < 7%	50	Brazil	median: 62	24	T2D population	4
Del Brutto et al. [19]	2014	Sleep quality	Low quality: PSQI > 5; high quality: PSQI ≤ 5	Glucose levels	FPG ≥ 100 mg/dL	635	Ecuador	59 ± 13	42	-	5
Demirtaş et al. [20]	2023	Sleep quality	Low quality: PSQI > 5; high quality: PSQI ≤ 5	Glucose levels	Adverse glycaemic control: HbA1c ≥ 7%; normal glycaemic control: HbA1c < 7%	103	Turkey	53.19 ± 10.03	46.6	Diabetic population	5
DePietro et al. [21]	2017	Sleep efficiency	Wrist-actigraphy	Diabetes	Clinical diagnosis	212	USA	63.9 ± 11.1	40.1	Diabetic population and non-diabetes population	5
Ding et al. [22]	2019	Insomnia	Insomnia: ISI > 14; no insomnia: ISI ≤ 14	Glucose levels	HbA1c (%)	3,749	China	Insomnia population: 54.2 ± 8.3; non-insomnia population: 54.3 ± 8.6	57.3	T2D population	5

First Author	Year	Exposure	Indicator	Outcome	Indicator	Sample size	Details of participants				NOS score
							Region	Age*	Gender (male, %)	Diabetes status	
Dong et al. [23]	2023	Insomnia	Self-reported	Diabetes	Clinical diagnosis	3,382	China	Median: 41.0	89.92	Diabetic population and non-diabetes population	5
Fukui et al. [24]	2012	Sleep quality	Low quality: PSQI > 5; high quality: PSQI ≤ 5	T2D	Clinical diagnosis	563	Japan	T2D population: 63.8 ± 10.0; non-diabetes population: 63.3 ± 4.1	100	T2D population and non-diabetes population	5
Gabbs et al. [25]	2022	Sleep quality	PSQI	T2D	Clinical diagnosis	240	Canada	T2D population: 15.11 ± 2.46; non-diabetes population: 16.31 ± 2.86	37.08	T2D population and non-diabetes population	6
Gara et al. [26]	2019	Sleep quality	Low quality: PSQI > 5; high quality: PSQI ≤ 5	Glucose levels	HbA1c (%)	444	India	53.33 ± 10.49	42.34	T2D population	5
Gozashti et al. [27]	2016	Sleep quality	Low quality: PSQI > 5; high quality: PSQI ≤ 5	Glucose levels	HbA1c (%)	118	Iran	58 ± 11	76.27	T2D population	6
Haliloglu et al. [28]	2020	Sleep quality	PSQI	T2D	Clinical diagnosis	78	Turkey	T2D population: 53.8 ± 12.5; non-diabetes population: 45.7 ± 14.1	35.9	T2D population and non-diabetes population	5
Hayashino et al. [29]	2013	Sleep quality	Low quality: PSQI > 5; high quality: PSQI ≤ 5	Glucose levels	HbA1c (%)	1,513	Japan	63.7 ± 12.6	50.6	T1D population and T2D population	6
Hilmisson et al. [30]	2019	Sleep quality	Low quality: Sleep quality index (SQI) <60; high quality: SQI ≥ 80	Glucose levels	FPG (mg/dL)	72	USA	Low sleep quality population: 6.3 ± 1.2; high sleep quality population: 6.3 ± 1.0	53.53	-	7

First Author	Year	Exposure	Indicator	Outcome	Indicator	Sample size	Details of participants				NOS score
							Region	Age*	Gender (male, %)	Diabetes status	
Huang et al. [31]	2017	Sleep quality	Low quality: PSQI > 7; high quality: PSQI ≤ 7	Glucose levels	HbA1c (%)	81	China	Low sleep quality population: 66.2 ± 8.8; high sleep quality population: 65.6 ± 10.3	53.33	T2D population	6
Hung et al. [32]	2013	Sleep quality	PSQI	Glucose levels	IFG: FPG of 5.6–7.0 mmol/L and 2-h post load glucose <7.8 mmol/L; otherwise, no IFG:	1,335	China	IFG population: 51.4 ± 8.8; non-IFG population: 50.0 ± 8.1	60.79	Non-diabetes population	5
Hur et al. [33]	2020	Sleep efficiency	Wrist-actigraphy	T2D	Clinical diagnosis	60	Korea	T2D population: 47.3 ± 10.68; non-diabetes population: 42.9 ± 12.45	53.33	T2D population and non-diabetes population	6
Imes et al. [34]	2022	Insomnia	Insomnia: ISI ≥ 10; no insomnia: ISI < 10	Glucose levels	HbA1c (%)	149	USA	56.3 ± 10.5	51.4	T2D population	6
Inoue et al. [35]	2021	Insomnia	Questionnaire	Diabetes	Clinical diagnosis	7,324	Japan	Diabetes population: 62.0 ± 15.1; non-diabetes population: 44.7 ± 16.5	49.66	Diabetic population and non-diabetes population	5
Ishibashi et al. [36]	2020	Sleep quality	PSQI	T2D	Clinical diagnosis	178	Japan	T2D population: 48.6 ± 0.7; non-diabetes population: 48.6 ± 1.2	62.92	T2D population and non-diabetes population	6
Jain et al. [37]	2012	Insomnia	-	Glucose levels	HbA1c (%)	19	USA	Insomnia population: 51.6 ± 4.6; non-insomnia population: 54.2 ± 2.4	21.05	T2D population	5

First Author	Year	Exposure	Indicator	Outcome	Indicator	Sample size	Details of participants				NOS score
							Region	Age*	Gender (male, %)	Diabetes status	
Jemere et al. [38]	2019	Sleep quality	Low quality: PSQI > 5; high quality: PSQI ≤ 5	Glucose levels	Adverse glycaemic control: FPG > 130 mg/dL or < 70 mg/dL; otherwise, normal adverse glycaemic control	99	Ethiopia	T2D population: 50.14 ± 11.3; non-diabetes population: 49.9 ± 9.7	52.53	T2D population and non-diabetes population	6
Kachi et al. [39]	2011	Insomnia	Questionnaire	Glucose levels	Adverse glycaemic control: HbA1c ≥ 6%; normal glycaemic control: HbA1c < 6%	1,042	Japan	43.9 ± 10.1	100	-	4
Keckeis et al. [40]	2010	Insomnia	-	Glucose levels	Adverse glycaemic control: HbA1c, ≥ 5.5% and FPG ≥ 100 mg/dL; otherwise, normal glycaemic control	52	Germany	Insomnia population: 49.1 ± 9.7; non-insomnia population: 46.8 ± 7.7	46.3	Non-diabetes population	4
Keskin et al. [41]	2015	Sleep quality	Low quality: PSQI ≥ 5; high quality: PSQI < 5	Glucose levels	Adverse glycaemic control: HbA1c > 6.5%; normal glycaemic control: HbA1c ≤ 6.5%	564	Turkey	Median: 58	33.04	T2D population	6

First Author	Year	Exposure	Indicator	Outcome	Indicator	Sample size	Details of participants				NOS score
							Region	Age*	Gender (male, %)	Diabetes status	
Khakurel et al. [42]	2020	Sleep quality	PSQI (≥8, <8)	Glucose levels	Adverse glycaemic control: HbA1c ≥ 7%; normal glycaemic control: HbA1c < 7%	208	Nepal	55.36 ± 10.58	45.2	T2D population	5
Kita et al. [43]	2012	Sleep quality	Athens Insomnia Scale (AIS)	Diabetes	Clinical diagnosis	2,862	Japan	Diabetes population: 50.3 ± 4.0; non-diabetes population: 46.2 ± 6.1	79.3	Diabetic population and non-diabetes population	6
Kostkova et al. [44]	2018	Sleep efficiency	Video polysomnography	T1D	Clinical diagnosis	104	Slovakia	T1D population: 14.4 ± 2.5; non-diabetes population: 13.9 ± 0.33	40.38	T1D population and non-diabetes population	6
Lee et al. [45]	2016	Sleep quality	Low quality: PSQI ≥ 5; high quality: PSQI < 5	Glucose levels	IFG: 110 mg/dL ≤ FPG ≤ 125 mg/dL; otherwise, no IFG	463	China	57.0 ± 10.2	45.6	Non-diabetes population	6
Lou et al. [46]	2014	Sleep quality	Low quality: PSQI > 5; high quality: PSQI ≤ 5	Glucose levels	IFG: 110 mg/dL ≤ FPG ≤ 125 mg/dL; otherwise, no IFG	15,145	China	47.6 ± 15.1	49.9	Non-diabetes population	6
Lou et al. [47]	2012	Sleep quality	Self-reported sleep quality	T2D	Clinical diagnosis	16,893	China	45.1 ± 14.4	45.6	T2D population and non-diabetes population	6

First Author	Year	Exposure	Indicator	Outcome	Indicator	Sample size	Details of participants				NOS score
							Region	Age*	Gender (male, %)	Diabetes status	
Lou et al. [48]	2015	Sleep quality	Low quality: PSQI > 7; high quality: PSQI ≤ 7	Glucose levels	Adverse glycaemic control: HbA1c ≥ 6.5%; normal glycaemic control: HbA1c < 6.5%	944	China	64.1 ± 10.2	38.7	T2D population	6
Lou et al. [49]	2015	Sleep quality	Questionnaire	T2D	Clinical diagnosis	4,213	China	44.8 ± 14.7	45.4	T2D population and non-diabetes population	6
Martyn-Nemeth et al. [50]	2018	Sleep quality	Low quality: PSQI > 5; high quality: PSQI ≤ 5	Glucose levels	HbA1c (%)	48	USA	27.0 ± 5.8	37	T1D population	6
Mehrdad et al. [51]	2021	Sleep quality	Low quality: PSQI > 5; high quality: PSQI ≤ 5	Glucose levels	HbA1c (%)	266	Iran	47.00 ± 19.04	34.6	T1D population and T2D population	5
Meng et al. [52]	2015	Sleep quality	Low quality: PSQI < 7; high quality: PSQI ≥ 7	Glucose levels	HbA1c (%)	332	China	Low sleep quality population: 59.36 ± 9.39; high sleep quality population: 53.09 ± 13.60	56.63	T2D population	5
Mokhlesi et al. [53]	2019	Sleep quality	PSQI	T2D	Clinical diagnosis	962	USA	52.2 ± 9.5	54.6	T2D population and non-diabetes population	5
Mokhlesi et al. [54]	2019	Sleep quality	Sleep Disturbances Scale questionnaire, low quality: ≥52; high quality: <52	T2D	Clinical diagnosis	96	USA	14.1 ± 2.1	29.9	T2D population and non-diabetes population	6

First Author	Year	Exposure	Indicator	Outcome	Indicator	Sample size	Details of participants				NOS score
							**Region**	**Age***	**Gender (male, %)**	**Diabetes status**	
Narisawa et al. [55]	2017	Sleep quality	PSQI	T2D	Clinical diagnosis	1,244	Japan	T2D population: 56.8 ± 9.62; non-diabetes population: 56.1 ± 9.56	75.88	T2D population and non-diabetes population	6
Nefs et al. [56]	2015	Sleep quality	Low quality: PSQI > 5; high quality: PSQI ≤ 5	Glucose levels	HbA1c (%)	243	Netherlands	62 ± 9	54	T2D population	4
O et al. [57]	2023	Insomnia	Insomnia: ISI > 14; no insomnia: ISI ≤ 14	Glucose levels	HbA1c (%)	986	China	Insomnia population: 62.6 ± 2.5; non-insomnia population: 62.5 ± 2.6	58.14	T2D population	5
Osonoi et al. [58]	2015	Sleep quality	Low quality: PSQI > 5; high quality: PSQI ≤ 5	Glucose levels	HbA1c (%)	539	Japan	57.8±8.6	62.98	T2D population	5
Pan et al. [59]	2022	Insomnia	Insomnia: ISI > 14; no insomnia: ISI ≤ 14	Glucose levels	FPG (mg/dL)	118	China	Insomnia population: 72.33 ± 5.95; non-insomnia population: 71.84 ± 6.24	51.69	-	5
Pillar et al. [60]	2003	Sleep efficiency	Polysomnography	T1D	Clinical diagnosis	30	Israel	T1D population: 12.6 ± 2.9; non-diabetes population: 13.3 ± 1.1	53.33	T1D population and non-diabetes population	6
Qin et al. [61]	2016	Sleep quality	Low quality: PSQI > 5; high quality: PSQI ≤ 5	Glucose levels	IFG: FPG ≥ 126 mg/dL; no IFG: FPG < 126 mg/dL	15,145	China	47.6 ± 15.1	49.9	-	6

First Author	Year	Exposure	Indicator	Outcome	Indicator	Sample size	Details of participants				NOS score
							Region	Age*	Gender (male, %)	Diabetes status	
Reutrakul et al. [62]	2011	Sleep quality	Low quality: PSQI > 5; high quality: PSQI ≤ 5	GDM	A 100 g oral glucose tolerance test during the second trimester of gestation	142	USA	28.5 ± 5.5	0	GDM population	3
Richa et al. [63]	2023	Sleep quality	PSQI (≥5, <5)	Glucose levels	Adverse glycaemic control: HbA1c ≥ 7%; normal glycaemic control: HbA1c < 7%	192	India	52.7 ± 10.3	53.13	T2D population	6
Rizza et al. [64]	2021	Sleep quality	Low quality: PSQI ≥ 5; high quality: PSQI < 5	Glucose levels	HbA1c (%)	273	Italy	Low sleep quality population: 38.7 ± 6.3; high sleep quality population: 36.9 ± 6.5	32.23	Non-diabetes population	5
Ruangchaisiwaw et et al. [65]	2024	Sleep quality	Low quality: PSQI > 5; high quality: PSQI ≤ 5	Glucose levels	Adverse glycaemic control: HbA1c ≥ 7%; normal glycaemic control: HbA1c < 7%	127	Thailand	66.2 ± 7.3	48.03	T2D population	5

First Author	Year	Exposure	Indicator	Outcome	Indicator	Sample size	Details of participants				NOS score
							Region	Age*	Gender (male, %)	Diabetes status	
Sakamoto et al. [66]	2018	Sleep quality	Low quality: PSQI ≥ 6; high quality: PSQI < 6	Glucose levels	Adverse glycaemic control: HbA1c > 7%; normal glycaemic control: HbA1c ≤ 7%	3,294	Japan	Median: 65	61.08	T2D population	5
Shamshirgaran et al. [67]	2017	Sleep quality	Low quality: PSQI > 5; high quality: PSQI ≤ 5	Glucose levels	Adverse glycaemic control: HbA1c ≥ 7%; normal glycaemic control: HbA1c < 7%	256	Iran	54.06 ± 9.09	29	T2D population	6
Silva-Costa et al. [68]	2020	Insomnia	Questionnaire	Glucose levels	Adverse glycaemic control: HbA1c ≥ 6.5%; normal glycaemic control: HbA1c < 6.5%	6,231	Brazil	Insomnia population: 56.3 ± 9.2; non-insomnia population: 55.6 ± 9.2	100	-	5
Simon et al. [69]	2018	Sleep efficiency	Wrist-actigraphy	Diabetes	Clinical diagnosis	22	USA	Diabetes population: 14.09 ± 2.2; non-diabetes population: 13.5 ± 2.3	40.91	Diabetic population and non-diabetes population	5
Suárez-Torres et al. [70]	2023	Insomnia	Insomnia: AIS >6; no insomnia: AIS ≤ 6	Glucose levels	Adverse glycaemic control: HbA1c ≥ 7%; normal glycaemic control: HbA1c < 7%	202	Mexico	Median: 53	29	T2D population	7

First Author	Year	Exposure	Indicator	Outcome	Indicator	Sample size	Details of participants				NOS score
							Region	Age*	Gender (male, %)	Diabetes status	
Sugano et al. [71]	2022	Insomnia	Questionnaire	Glucose levels	IFG: FPG ≥ 110 mg/dL; no IFG: FPG < 110 mg/dL	755	Japan	50.1 ± 7.2	100	-	5
Suteau et al. [72]	2020	Sleep quality	Low quality: PSQI > 5; high quality: PSQI ≤ 5	Glucose levels	HbA1c (%)	315	France	47 ± 15	46	T1D population	4
Telford et al. [73]	2019	Sleep quality	Low quality: PSQI > 5; high quality: PSQI ≤ 5	Glucose levels	HbA1c (%)	281	USA	61.9 ± 8.8	51.6	T2D population	4
Trento et al. [74]	2008	Sleep efficiency	Wrist-actigraphy	T2D	Clinical diagnosis	70	Italy	T2D population: 61.0 ± 4.9; non-diabetes population: 58.0 ± 9.7	61.43	T2D population and non-diabetes population	5
Tsai et al. [75]	2011	Sleep quality	Low quality: PSQI > 7; high quality: PSQI ≤ 7	Glucose levels	Adverse glycaemic control: HbA1c ≥ 7%; normal glycaemic control: HbA1c < 7%	46	China	60.11 ± 9.69	60.87	T2D population	4
Vargas et al. [76]	2021	Sleep quality	Low quality: PSQI ≥ 5; high quality: PSQI < 5	Glucose levels	FPG (mg/dL)	26	Chile	39.9 ± 11.94	0	-	5
Vézina-Im et al. [77]	2021	Insomnia	PSQI	Diabetes	Clinical diagnosis	151	Canada	Adults	19.9	T1D population, T2D population and non-diabetes population	7
Vgontzas et al. [78]	2009	Insomnia	A complaint of insomnia with a duration of at least 1 y	Glucose levels	FPG (mg/dL)	1,221	USA	-	48	T2D population and non-diabetes population	5

First Author	Year	Exposure	Indicator	Outcome	Indicator	Sample size	Details of participants				NOS score
							Region	Age*	Gender (male, %)	Diabetes status	
Wu et al. [79]	2023	Sleep quality	PSQI	PGDM or GDM	The 75-g OGTT method	1,335	China	GDM population: 31.73 ± 4.41; non-diabetes population: 28.27 ± 3.56	0	PGDM population, GDM population, and non-diabetes population	5
Xu et al. [80]	2019	Sleep quality	Low quality: PSQI > 7; high quality: PSQI ≤ 7	Glucose levels	Adverse glycaemic control: HbA1c ≥ 7%; normal glycaemic control: HbA1c < 7%	944	China	Range: 30-89	35.7	T2D population	6
Yang et al. [81]	2021	Sleep quality	Low quality: PSQI > 7; high quality: PSQI ≤ 7	Glucose levels	HbA1c (%)	70	China	50.1 ± 11.1	63.96	T2D population	5
Yoshida et al. [82]	2018	Insomnia	Low quality: PSQI ≥ 5.5; high quality: PSQI < 5.5	Glucose levels	HbA1c (%)	503	Japan	Insomnia population: 61.3 ± 13.6; non-insomnia population: 64.9 ± 11.9	58.05	T2D population	4
Zhu et al. [83]	2014	Sleep quality	Low quality: PSQI ≥ 8; high quality: PSQI < 8	Glucose levels	FPG (mmol/L)	206	China	57.23 ± 11.24	66	T2D population	6
Zubair et al. [84]	2018	Sleep quality	PSQI	T2D	Clinical diagnosis	100	Pakistan	50 ± 7	38	T2D population and non-diabetes population	5
Case-control studies											
Agyekum et al. [85]	2023	Sleep quality	PSQI	T2D	Clinical diagnosis	360	Ghana	T2D population: 52.0 ± 7.9; non-diabetes population: 48.6 ± 10.6	36.11	T2D population and non-diabetes population	8

First Author	Year	Exposure	Indicator	Outcome	Indicator	Sample size	Details of participants				NOS score
							Region	Age*	Gender (male, %)	Diabetes status	
Al-Musharaf et al. [86]	2022	Sleep quality	Low quality: PSQI > 5; high quality: PSQI ≤ 5	Glucose levels	FPG (mg/dL)	92	Saudi Arabia	21.1 ± 1.5	0	-	8
Bahadur et al. [87]	2022	Sleep quality	CSHQ	T1D	Clinical diagnosis	124	Turkey	T1D population: 8.81 ± 2.28; non-diabetes population: 8.14±2.3	38.71	T1D population and non-diabetes population	7
Henriksen et al. [88]	2023	Insomnia	Questionnaire	T2D	Clinical diagnosis	20,295	Norway	T2D population: 69.9 ± 9.8; non-diabetes population:65.3 ± 11.2	44.78	T2D population and non-diabetes population	9
van Dijk et al. [89]	2011	Sleep quality	PSQI	T1D	Clinical diagnosis	198	Netherlands	T1D population: 43.9 ± 1.3; non-diabetes population: 44.1 ± 1.3	55.56	T1D population and non-diabetes population	8
Cohort studies											
LeBlanc et al. [90]	2018	Insomnia	physician-entered diagnosis of insomnia or use medications for sleep problems	T2D	Clinical diagnosis	81,233	USA	57.5 ± 13.6	46.37	Non-diabetes population	7
Zou et al. [91]	2022	Sleep quality	Low quality: PSQI > 5; high quality: PSQI ≤ 5	GDM	a 75 g oral glucose tolerance test	3,329	China	GDM population: 30.08 ± 4.28; non-diabetes population: 28.43 ± 3.95	0	Non-diabetes population	6

PSQI – Pittsburgh Sleep Quality Index, SDSC – The Sleep Disturbance Scale for Children, CSHQ – The Children's Sleep Habits Questionnaire, ISI – Insomnia Severity Index, AIS – Athens Insomnia Scale, HbA1c – haemoglobin A1c, FPG – fasting plasma glucose, IFG – impaired fasting glucose, T1D – type 1 diabetes, T2D – type 2 diabetes, GDM – gestational diabetes mellitus, PGDM – pregestational diabetes mellitus, NOS – The Newcastle-Ottawa Scale.

* If there is no specific explanation, age is presented as mean (±standard deviation) by default.

Table S3. The details of quality assessment

Study	Selection	Comparability	Exposure/outcome	Total score
Cross-sectional studies				
Abdu et al. [1]	111	11	11	7
Adler et al. [2]	011	11	11	6
Al-Musharaf et al. [3]	011	11	11	6
Aribas et al. [4]	011	11	11	6
Azuma et al. [5]	010	01	11	4
Bani-Issa et al. [6]	101	11	11	6
Barakat et al. [7]	001	00	11	3
Barikani et al. [8]	011	11	11	6
Barone et al. [9]	011	11	11	6
Bener et al. [10]	111	11	11	7
Caruso et al. [11]	101	11	11	6
Cho et al. [12]	101	01	11	5
Clark et al. [13]	101	01	11	5
Colbay et al. [14]	011	11	11	6
Çömlek et al. [15]	011	11	11	6
Corrado et al. [16]	011	11	11	6
Culver at al.[17]	011	11	11	6
Cunha et al. [18]	111	00	10	4
Del Brutto et al. [19]	011	01	11	5
Demirtaş et al. [20]	011	01	11	5
DePietro et al. [21]	011	01	11	5
Ding et al. [22]	011	01	11	5
Dong et al. [23]	110	01	11	5

Study	Selection	Comparability	Exposure/outcome	Total score
Fukui et al. [24]	011	01	11	5
Gara et al. [26]	011	01	11	5
Gabbs et al. [25]	011	11	11	6
Gozashti et al. [27]	011	11	11	6
Haliloglu et al. [28]	011	01	11	5
Hayashino et al. [29]	011	11	11	6
Hilmisson et al. [30]	111	11	11	7
Huang et al. [31]	011	11	11	6
Hung et al. [32]	011	01	11	5
Hur et al. [33]	011	11	11	6
Imes et al. [34]	111	01	11	6
Inoue et al. [35]	110	01	11	5
Ishibashi et al. [36]	011	11	11	6
Jain et al. [37]	010	01	11	4
Jemere et al. [38]	011	11	11	6
Kachi et al. [39]	010	01	11	4
Keckeis et al. [40]	010	01	11	4
Keskin et al. [41]	011	11	11	6
Khakurel et al. [42]	101	10	11	5
Kita et al. [43]	111	01	11	6
Kostkova et al. [44]	011	11	11	6
Lee et al. [45]	011	11	11	6
Lou et al. [46]	111	01	11	6
Lou et al. [47]	110	11	11	6
Lou et al. [48]	111	01	11	6

Study	Selection	Comparability	Exposure/outcome	Total score
Lou et al. [49]	111	01	11	6
Martyn-Nemeth et al. [50]	011	11	11	6
Mehrdad et al. [51]	101	01	11	5
Meng et al. [52]	011	01	11	5
Mokhlesi et al. [53]	011	01	11	5
Mokhlesi et al. [54]	011	11	11	6
Narisawa et al. [55]	011	11	11	6
Nefs et al. [56]	001	01	11	4
O et al. [57]	011	01	11	5
Osonoi et al. [58]	011	01	11	5
Pan et al. [59]	001	11	11	5
Pillar et al. [60]	011	11	11	6
Qin et al. [61]	111	01	11	6
Reutrakul et al. [62]	001	00	11	3
Richa et al. [63]	111	01	11	6
Rizza et al. [64]	011	01	11	5
Ruangchaisiwawet et al. [65]	011	01	11	5
Sakamoto et al. [66]	011	01	11	5
Shamshirgaran et al. [67]	011	11	11	6
Silva-Costa et al. [68]	110	01	11	5
Simon et al. [69]	001	11	11	5
Suárez-Torres et al. [70]	111	11	11	7
Sugano et al. [71]	101	01	11	5
Suteau et al. [72]	001	01	11	4
Telford et al. [73]	001	01	11	4

Study	Selection	Comparability	Exposure/outcome	Total score
Trento et al. [74]	001	11	11	5
Tsai et al. [75]	001	01	11	4
Vargas et al. [76]	001	11	11	5
Vézina-Im et al. [77]	111	11	11	7
Vgontzas et al. [78]	110	01	11	5
Wu et al. [79]	101	01	11	5
Xu et al. [80]	111	01	11	6
Yang et al. [81]	001	11	11	5
Yoshida et al. [82]	001	01	11	4
Zhu et al. [83]	111	01	11	6
Zubair et al. [84]	011	01	11	5
Case-control studies				
Agyekum et al. [85]	1111	01	111	8
Al-Musharaf et al. [86]	1011	11	111	8
Bahadur et al. [87]	1001	11	111	7
Henriksen et al. [88]	1111	11	111	9
van Dijk et al. [89]	1011	11	111	8
Cohort studies				
LeBlanc et al. [90]	1111	00	111	7
Zou et al. [91]	0111	00	111	6

References

1 Abdu Y, Naja S, Mohamed Ibrahim MI, Abdou M, Ahmed R, Elhag S, et al. Sleep Quality Among People with Type 2 Diabetes Mellitus During COVID-19 Pandemic: Evidence from Qatar's National Diabetes Center. Diabetes, metabolic syndrome and obesity : targets and therapy. 2023;16:2803-12.

2 Adler A, Gavan MY, Tauman R, Phillip M, Shalitin S. Do children, adolescents, and young adults with type 1 diabetes have increased prevalence of sleep disorders? Pediatric diabetes. 2017;18:450-8.

3 Al-Musharaf S, Albedair B, Alfawaz W, Aldhwayan M, Aljuraiban GS. The Relationships between Various Factors and Sleep Status: A Cross-Sectional Study among Healthy Saudi Adults. Nutrients. 2023;15:4090.

4 Aribas A, Kayrak M, Tekinalp M, Akilli H, Alibasic H, Yildirim S, et al. The relationship between serum asymmetric dimethylarginine levels and subjective sleep quality in normotensive patients with type 2 diabetes mellitus. The Korean journal of internal medicine. 2015;30:316-24.

5 Azuma T, Irie K, Watanabe K, Deguchi F, Kojima T, Obora A, et al. Association between Chewing Problems and Sleep among Japanese Adults. Int J Dent. 2019;2019:8196410.

6 Bani-Issa W, Al-Shujairi AM, Patrick L. Association between quality of sleep and health-related quality of life in persons with diabetes mellitus type 2. Journal of clinical nursing. 2018;27:1653-61.

7 Barakat S, Abujbara M, Banimustafa R, Batieha A, Ajlouni K. Sleep Quality in Patients With Type 2 Diabetes Mellitus. Journal of clinical medicine research. 2019;11:261-6.

8 Barikani A, Javadi M, Rafiei S. Sleep Quality and Blood Lipid Composition Among Patients with Diabetes. International journal of endocrinology and metabolism. 2019;17:e81062.

9 Barone MT, Wey D, Schorr F, Franco DR, Carra MK, Lorenzi Filho G, et al. Sleep and glycemic control in type 1 diabetes. Archives of endocrinology and metabolism. 2015;59:71-8.

10 Bener A, Al-Hamaq A, Agan AF, Öztürk M, Ömer A. Sleeping disturbances and predictor risk factors among type 2 diabetic mellitus patients. Annals of African medicine. 2020;19:230-6.

11 Caruso NC, Radovanovic B, Kennedy JD, Couper J, Kohler M, Kavanagh PS, et al. Sleep, executive functioning and behaviour in children and adolescents with type 1 diabetes. Sleep medicine. 2014;15:1490-9.

12 Cho MK, Kim MY. Associated factors with depression and sleep quality in T1DM patients: a cross-sectional descriptive study. BMC psychiatry. 2023;23:18.

13 Clark AJ, Dich N, Lange T, Jennum P, Hansen AM, Lund R, et al. Impaired sleep and allostatic load: cross-sectional results from the Danish Copenhagen Aging and Midlife Biobank. Sleep medicine. 2014;15:1571-8.

14 Colbay G, Cetin M, Colbay M, Berker D, Guler S. Type 2 diabetes affects sleep quality by disrupting the respiratory function. Journal of diabetes. 2015;7:664-71.
15 Çömlek F, Çelik H, Keskin B, Süt N, Dilek E, Tütüncüler F. Sleep Quality Assessment in Adolescents with and without Type 1 Diabetes Using the Pittsburg Sleep Quality Index. Indian journal of endocrinology and metabolism. 2021;25:202-5.
16 Corrado A, Scidà G, Vitale M, Caprio B, Costabile G, Annuzzi E, et al. Eating habits and sleep quality in individuals with type 1 diabetes on continuous glucose monitoring and insulin pump. Nutrition, metabolism, and cardiovascular diseases : NMCD. 2024;S0939-4753:00093-0.
17 Culver MN, Langan SP, Carreker J, Flatt AA, Ratchford SM, Grosicki GJ. Self-reported sleep quality is associated with central hemodynamics in healthy individuals. Sleep & breathing = Schlaf & Atmung. 2020;24:1083-8.
18 Cunha MC, Zanetti ML, Hass VJ. Sleep quality in type 2 diabetics. Revista latino-americana de enfermagem. 2008;16:850-5.
19 Del Brutto OH, Mera RM, Zambrano M, Del Brutto VJ, Castillo PR. Association between sleep quality and cardiovascular health: a door-to-door survey in rural Ecuador. Environ Health Prev Med. 2014;19:234-7.
20 Demirtaş H, Dolu İ. The prevalence of poor sleep quality and its association with the risk of obstructive sleep apnea and restless legs syndrome in diabetic patients treated with cyanoacrylate glue for varicose veins. Sleep & breathing = Schlaf & Atmung. 2023;27:745-55.
21 DePietro RH, Knutson KL, Spampinato L, Anderson SL, Meltzer DO, Van Cauter E, et al. Association Between Inpatient Sleep Loss and Hyperglycemia of Hospitalization. Diabetes care. 2017;40:188-93.
22 Ding C, Zhang J, Lau ESH, Luk AOY, So WY, Ma RCW, et al. Gender differences in the associations between insomnia and glycemic control in patients with type 2 diabetes: a cross-sectional study. Sleep. 2019;42:zsz014.
23 Dong C, Liu H, Yang B, Pan J, Tang L, Zeng H, et al. Circadian rhythm sleep-wake disorders and the risk of dyslipidemia among railway workers in southwest China: A cross-sectional study. Chronobiology international. 2023;40:734-43.
24 Fukui M, Tanaka M, Toda H, Okada H, Ohnishi M, Mogami S, et al. Andropausal symptoms in men with Type 2 diabetes. Diabetic medicine : a journal of the British Diabetic Association. 2012;29:1036-42.
25 Gabbs MHJ, Dart AB, Woo MR, Pinto T, Wicklow BA. Poor Sleep, Increased Stress and Metabolic Comorbidity in Adolescents and Youth With Type 2 Diabetes. Canadian journal of diabetes. 2022;46:142-9.
26 Gara DR, Panda K, Vanamali D. Subjective sleep quality in type-2 diabetics. Indian journal of physiology and pharmacology. 2019;63:155-9.
27 Gozashti MHM, Eslami NM, Radfar MHM, Pakmanesh HM. Sleep Pattern, Duration and Quality in Relation with Glycemic Control in People with Type 2 Diabetes Mellitus. Iranian journal of medical sciences. 2016;41:531-8.

28 Haliloglu Ö, Tütüncü M, Sahin S, Polat Korkmaz Ö, Özer MD, Osar Siva Z. Fatigue is Related to Insulin Use by Acting Via Depressive Mood in Patients with Diabetes Mellitus. Turk J Endocrinol Metab. 2020;24:29-37.

29 Hayashino Y, Tsujii S, Ishii H. High frequency of non-nocturnal hypoglycemia was associated with poor sleep quality measure by Pittsburg Sleep Quality Index in patients with diabetes receiving insulin therapy: Diabetes Distress and Care Registry at Tenri (DDCRT 4). Experimental and clinical endocrinology & diabetes : official journal, German Society of Endocrinology [and] German Diabetes Association. 2013;121:628-34.

30 Hilmisson H, Lange N, Magnusdottir S. Objective sleep quality and metabolic risk in healthy weight children results from the randomized Childhood Adenotonsillectomy Trial (CHAT). Sleep & breathing = Schlaf & Atmung. 2019;23:1197-208.

31 Huang Y, Wang H, Li Y, Tao X, Sun J. Poor Sleep Quality Is Associated with Dawn Phenomenon and Impaired Circadian Clock Gene Expression in Subjects with Type 2 Diabetes Mellitus. International journal of endocrinology. 2017;2017:4578973.

32 Hung HC, Yang YC, Ou HY, Wu JS, Lu FH, Chang CJ. The relationship between impaired fasting glucose and self-reported sleep quality in a Chinese population. Clinical endocrinology. 2013;78:518-24.

33 Hur MH, Lee MK, Seong K, Hong JH. Deterioration of Sleep Quality According to Glycemic Status. Diabetes & metabolism journal. 2020;44:679-86.

34 Imes CC, Bizhanova Z, Sereika SM, Korytkowski MT, Atwood CW, Jr., Burke LE, et al. Metabolic outcomes in adults with type 2 diabetes and sleep disorders. Sleep & breathing = Schlaf & Atmung. 2022;26:339-46.

35 Inoue K, Semba E, Yamakawa T, Terauchi Y. Associations of impaired glucose tolerance and sleep disorders with mortality among the US general population. BMJ open diabetes research & care. 2021;9:e002047.

36 Ishibashi F, Tavakoli M. Thinning of Macular Neuroretinal Layers Contributes to Sleep Disorder in Patients With Type 2 Diabetes Without Clinical Evidences of Neuropathy and Retinopathy. Frontiers in endocrinology. 2020;11:69.

37 Jain SK, Kahlon G, Morehead L, Lieblong B, Stapleton T, Hoeldtke R, et al. The effect of sleep apnea and insomnia on blood levels of leptin, insulin resistance, IP-10, and hydrogen sulfide in type 2 diabetic patients. Metabolic syndrome and related disorders. 2012;10:331-6.

38 Jemere T, Mossie A, Berhanu H, Yeshaw Y. Poor sleep quality and its predictors among type 2 diabetes mellitus patients attending Jimma University Medical Center, Jimma, Ethiopia. BMC research notes. 2019;12:488.

39 Kachi Y, Nakao M, Takeuchi T, Yano E. Association between insomnia symptoms and hemoglobin A1c level in Japanese men. PloS one. 2011;6:e21420.

40 Keckeis M, Lattova Z, Maurovich-Horvat E, Beitinger PA, Birkmann S, Lauer CJ, et al. Impaired glucose tolerance in sleep disorders. PloS one. 2010;5:e9444.

41 Keskin A, Ünalacak M, Bilge U, Yildiz P, Güler S, Selçuk EB, et al. Effects of Sleep Disorders on Hemoglobin A1c Levels in Type 2 Diabetic Patients. Chinese medical journal. 2015;128:3292-7.

42 Khakurel G, Shakya D, Chalise P, Chalise S. Association of Subjective Sleep Quality and Glycemic Level in Patients with Type 2 Diabetes Mellitus: A cross sectional study. Kathmandu University medical journal (KUMJ). 2020;18:107-10.
43 Kita T, Yoshioka E, Satoh H, Saijo Y, Kawaharada M, Okada E, et al. Short sleep duration and poor sleep quality increase the risk of diabetes in Japanese workers with no family history of diabetes. Diabetes care. 2012;35:313-8.
44 Kostkova M, Durdik P, Ciljakova M, Vojtkova J, Sujanska A, Pozorciakova K, et al. Short-term metabolic control and sleep in children and adolescents with type 1 diabetes mellitus. Journal of diabetes and its complications. 2018;32:580-5.
45 Lee JA, Sunwoo S, Kim YS, Yu BY, Park HK, Jeon TH, et al. The Effect of Sleep Quality on the Development of Type 2 Diabetes in Primary Care Patients. Journal of Korean medical science. 2016;31:240-6.
46 Lou P, Chen P, Zhang L, Zhang P, Chang G, Zhang N, et al. Interaction of sleep quality and sleep duration on impaired fasting glucose: a population-based cross-sectional survey in China. BMJ open. 2014;4:e004436.
47 Lou P, Chen P, Zhang L, Zhang P, Yu J, Zhang N, et al. Relation of sleep quality and sleep duration to type 2 diabetes: a population-based cross-sectional survey. BMJ open. 2012;2:e000956.
48 Lou P, Qin Y, Zhang P, Chen P, Zhang L, Chang G, et al. Association of sleep quality and quality of life in type 2 diabetes mellitus: a cross-sectional study in China. Diabetes research and clinical practice. 2015;107:69-76.
49 Lou P, Zhang P, Zhang L, Chen P, Chang G, Zhang N, et al. Effects of sleep duration and sleep quality on prevalence of type 2 diabetes mellitus: A 5-year follow-up study in China. Diabetes research and clinical practice. 2015;109:178-84.
50 Martyn-Nemeth P, Phillips SA, Mihailescu D, Farabi SS, Park C, Lipton R, et al. Poor sleep quality is associated with nocturnal glycaemic variability and fear of hypoglycaemia in adults with type 1 diabetes. Journal of advanced nursing. 2018;74:2373-80.
51 Mehrdad M, Azarian M, Sharafkhaneh A, Alavi A, Zare R, Hassanzadeh Rad A, et al. Association Between Poor Sleep Quality and Glycemic Control in Adult Patients with Diabetes Referred to Endocrinology Clinic of Guilan: A Cross-sectional Study. International journal of endocrinology and metabolism. 2022;20:e118077.
52 Meng LL, Tang YZ, Ni CL, Yang M, Song HN, Wang G, et al. Impact of inflammatory markers on the relationship between sleep quality and incident cardiovascular events in type 2 diabetes. Journal of diabetes and its complications. 2015;29:882-6.
53 Mokhlesi B, Temple KA, Tjaden AH, Edelstein SL, Nadeau KJ, Hannon TS, et al. The association of sleep disturbances with glycemia and obesity in youth at risk for or with recently diagnosed type 2 diabetes. Pediatric diabetes. 2019;20:1056-63.
54 Mokhlesi B, Temple KA, Tjaden AH, Edelstein SL, Utzschneider KM, Nadeau KJ, et al. Association of Self-Reported Sleep and Circadian Measures With

Glycemia in Adults With Prediabetes or Recently Diagnosed Untreated Type 2 Diabetes. Diabetes care. 2019;42:1326-32.
55 Narisawa H, Komada Y, Miwa T, Shikuma J, Sakurai M, Odawara M, et al. Prevalence, symptomatic features, and factors associated with sleep disturbance/insomnia in Japanese patients with type-2 diabetes. Neuropsychiatric disease and treatment. 2017;13:1873-80.
56 Nefs G, Donga E, van Someren E, Bot M, Speight J, Pouwer F. Subjective sleep impairment in adults with type 1 or type 2 diabetes: Results from Diabetes MILES--The Netherlands. Diabetes research and clinical practice. 2015;109:466-75.
57 O CK, Siu BW, Leung VW, Lin YY, Ding CZ, Lau ES, et al. Association of insomnia with incident chronic cognitive impairment in older adults with type 2 diabetes mellitus: A prospective study of the Hong Kong Diabetes Register. Journal of diabetes and its complications. 2023;37:108598.
58 Osonoi Y, Mita T, Osonoi T, Saito M, Tamasawa A, Nakayama S, et al. Poor sleep quality is associated with increased arterial stiffness in Japanese patients with type 2 diabetes mellitus. BMC endocrine disorders. 2015;15:29.
59 Pan XL, Nie L, Zhao SY, Zhang XB, Zhang S, Su ZF. The Association Between Insomnia and Atherosclerosis: A Brief Report. Nature and science of sleep. 2022;14:443-8.
60 Pillar G, Schuscheim G, Weiss R, Malhotra A, McCowen KC, Shlitner A, et al. Interactions between hypoglycemia and sleep architecture in children with type 1 diabetes mellitus. The Journal of pediatrics. 2003;142:163-8.
61 Qin Y, Lou PA, Chen PP, Zhang L, Zhang P, Chang GQ, et al. Interaction of poor sleep quality, family history of type 2 diabetes, and abdominal obesity on impaired fasting glucose: a population-based cross-sectional survey in China. Int J Diabetes Dev Ctries. 2016;36:277-82.
62 Reutrakul S, Zaidi N, Wroblewski K, Kay HH, Ismail M, Ehrmann DA, et al. Sleep disturbances and their relationship to glucose tolerance in pregnancy. Diabetes care. 2011;34:2454-7.
63 Richa DR, Datta N, Raj V, Kumar R, Venugopal V. Sleep Quality in Type 2 Diabetes Mellitus Patients Attending a Tertiary Care Hospital in West Bengal: A Cross-Sectional Study. Cureus. 2023;15:e46163.
64 Rizza S, Luzi A, Mavilio M, Ballanti M, Massimi A, Porzio O, et al. Alterations in Rev-ERBα/BMAL1 ratio and glycated hemoglobin in rotating shift workers: the EuRhythDia study. Acta diabetologica. 2021;58:1111-7.
65 Ruangchaisiwawet A, Bankhum N, Tanasombatkul K, Phinyo P, Yingchankul N. Prevalence and the association between clinical factors and Diabetes-Related Distress (DRD) with poor glycemic control in patients with type 2 diabetes: A Northern Thai cross-sectional study. PloS one. 2023;18:e0294810.
66 Sakamoto R, Yamakawa T, Takahashi K, Suzuki J, Shinoda MM, Sakamaki K, et al. Association of usual sleep quality and glycemic control in type 2 diabetes in Japanese: A cross sectional study. Sleep and Food Registry in Kanagawa (SOREKA). PloS one. 2018;13:e0191771.
67 Shamshirgaran SM, Ataei J, Malek A, Iranparvar-Alamdari M, Aminisani N. Quality of sleep and its determinants among people with type 2 diabetes mellitus

in Northwest of Iran. World J Diabetes. 2017;8:358-64.
68 Silva-Costa A, Rotenberg L, Nobre AA, Chor D, Aquino EM, Melo EC, et al. Sex differences in the association between self-reported sleep duration, insomnia symptoms and cardiometabolic risk factors: cross-sectional findings from Brazilian longitudinal study of adult health. Arch Public Health. 2020;78:48.
69 Simon SL, Vigers T, Campbell K, Pyle L, Branscomb R, Nadeau KJ, et al. Reduced insulin sensitivity is correlated with impaired sleep in adolescents with cystic fibrosis. Pediatric diabetes. 2018;19:1183-90.
70 Suárez-Torres I, García-García F, Morales-Romero J, Melgarejo-Gutiérrez M, Demeneghi-Marini VP, Luna-Ceballos RI, et al. Poor quality of sleep in Mexican patients with type 2 diabetes and its association with lack of glycemic control. Primary care diabetes. 2023;17:155-60.
71 Sugano Y, Miyachi T, Ando T, Iwata T, Yamanouchi T, Mishima K, et al. Diabetes and anxiety were associated with insomnia among Japanese male truck drivers. Sleep medicine. 2022;90:102-8.
72 Suteau V, Saulnier PJ, Wargny M, Gonder-Frederick L, Gand E, Chaillous L, et al. Association between sleep disturbances, fear of hypoglycemia and psychological well-being in adults with type 1 diabetes mellitus, data from cross-sectional VARDIA study. Diabetes research and clinical practice. 2020;160:107988.
73 Telford O, Diamantidis CJ, Bosworth HB, Patel UD, Davenport CA, Oakes MM, et al. The relationship between Pittsburgh Sleep Quality Index subscales and diabetes control. Chronic illness. 2019;15:210-9.
74 Trento M, Broglio F, Riganti F, Basile M, Borgo E, Kucich C, et al. Sleep abnormalities in type 2 diabetes may be associated with glycemic control. Acta diabetologica. 2008;45:225-9.
75 Tsai YW, Kann NH, Tung TH, Chao YJ, Lin CJ, Chang KC, et al. Impact of subjective sleep quality on glycemic control in type 2 diabetes mellitus. Family practice. 2012;29:30-5.
76 Vargas CA, Guzmán-Guzmán IP, Caamaño-Navarrete F, Jerez-Mayorga D, Chirosa-Ríos LJ, Delgado-Floody P. Syndrome Metabolic Markers, Fitness and Body Fat Is Associated with Sleep Quality in Women with Severe/Morbid Obesity. International journal of environmental research and public health. 2021;18:9294.
77 Vézina-Im LA, Morin CM, Desroches S. Sleep, Diet and Physical Activity Among Adults Living With Type 1 and Type 2 Diabetes. Canadian journal of diabetes. 2021;45:659-65.
78 Vgontzas AN, Liao D, Pejovic S, Calhoun S, Karataraki M, Bixler EO. Insomnia with objective short sleep duration is associated with type 2 diabetes: A population-based study. Diabetes care. 2009;32:1980-5.
79 Wu QZ, Meng ZY, Liu Q, Zhang LL, Mao BH, Wang C, et al. Sleep quality in women with diabetes in pregnancy: a single-center retrospective study. BMC

Pregnancy Childbirth. 2023;23:597.
80 Xu C, Zhang P, Xiang Q, Chang G, Zhang M, Zhang L, et al. Relationship between subjective sleep disturbances and glycaemia in Chinese adults with type 2 diabetes: findings from a 1.5-year follow-up study. Scientific reports. 2019;9:14276.
81 Yang Y, Zhao LH, Li DD, Xu F, Wang XH, Lu CF, et al. Association of sleep quality with glycemic variability assessed by flash glucose monitoring in patients with type 2 diabetes. Diabetology & metabolic syndrome. 2021;13:102.
82 Yoshida K, Otaka H, Murakami H, Nakayama H, Murabayashi M, Mizushiri S, et al. Association between insomnia and coping style in Japanese patients with type 2 diabetes mellitus. Neuropsychiatric disease and treatment. 2018;14:1803-9.
83 Zhu B-Q, Li X-M, Wang D, Yu X-F. Sleep quality and its impact on glycaemic control in patients with type 2 diabetes mellitus. International Journal of Nursing Sciences. 2014;1:260-5.
84 Zubair U, Majid F, Siddiqui AA, Zubair Z. Sleep Abnormalities Among Patients With and Without Diabetes Using Pittsburg Sleep Quality Index and Epworth Sleepiness Scale. Cureus. 2018;10:e2151.
85 Agyekum JA, Gyamfi T, Yeboah K. Depression, poor sleep quality, and diabetic control in type 2 diabetes patients at Sunyani Regional Hospital, Ghana: a case-control study. Middle East Curr Psychiatry. 2023;30:45.
86 Al-Musharaf S, AlAjllan L, Aljuraiban G, AlSuhaibani M, Alafif N, Hussain SD. Nutritional Biomarkers and Factors Correlated with Poor Sleep Status among Young Females: A Case-Control Study. Nutrients. 2022;14:2898.
87 Bahadur EI, Özalkak S, Özdemir AA, Çetinkaya S, Özmert EN. Sleep disorder and behavior problems in children with type 1 diabetes mellitus. Journal of pediatric endocrinology & metabolism : JPEM. 2022;35:29-38.
88 Henriksen RE, Nilsen RM, Strandberg RB. Loneliness increases the risk of type 2 diabetes: a 20 year follow-up - results from the HUNT study. Diabetologia. 2023;66:82-92.
89 van Dijk M, Donga E, van Dijk JG, Lammers GJ, van Kralingen KW, Dekkers OM, et al. Disturbed subjective sleep characteristics in adult patients with long-standing type 1 diabetes mellitus. Diabetologia. 2011;54:1967-76.
90 LeBlanc ES, Smith NX, Nichols GA, Allison MJ, Clarke GN. Insomnia is associated with an increased risk of type 2 diabetes in the clinical setting. BMJ open diabetes research & care. 2018;6:e000604.
91 Zou J, Wei Q, Ye P, Shi Y, Zhang Y, Shi H. Effects of Gestational Sleep Patterns and Their Changes on Maternal Glycemia and Offspring Physical Growth in Early Life. Nutrients. 2022;14:3390.

Figure S1. The meta-analysis comparing the occurrence of low sleep quality between groups of adverse/normal glycaemic control

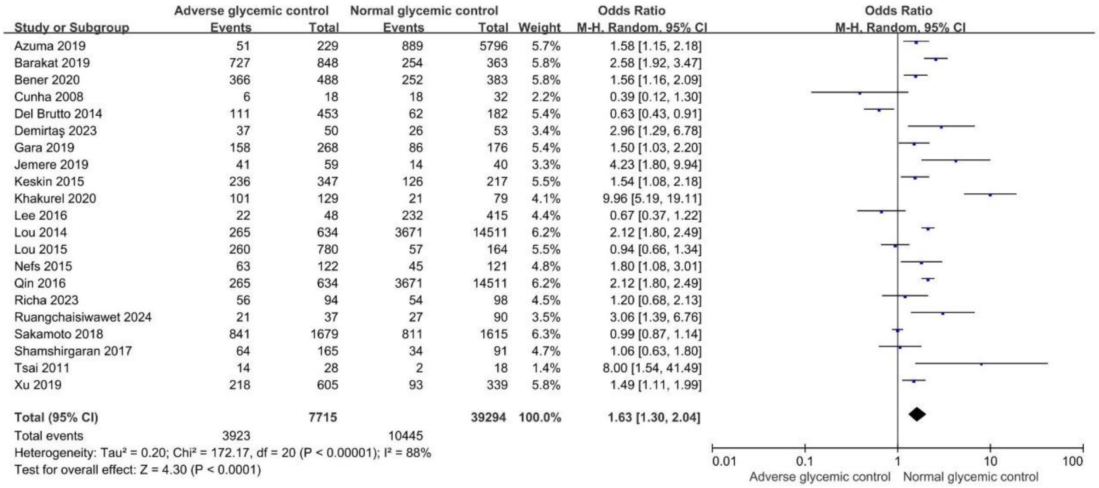

Figure S2. The meta-analysis comparing the occurrence of insomnia between groups of adverse/normal glycaemic control

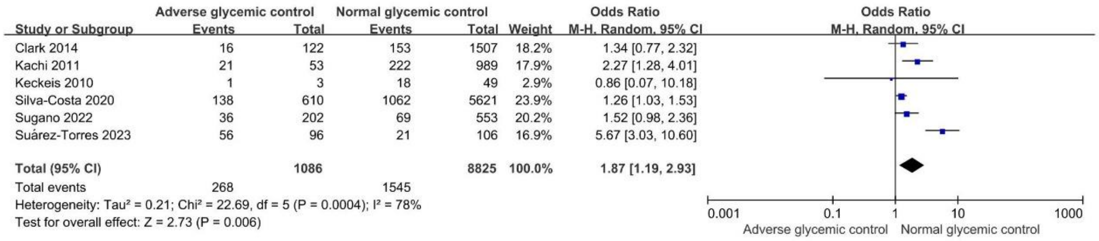

Figure S3. The meta-analysis comparing the occurrence of low sleep quality between groups with/without diabetes

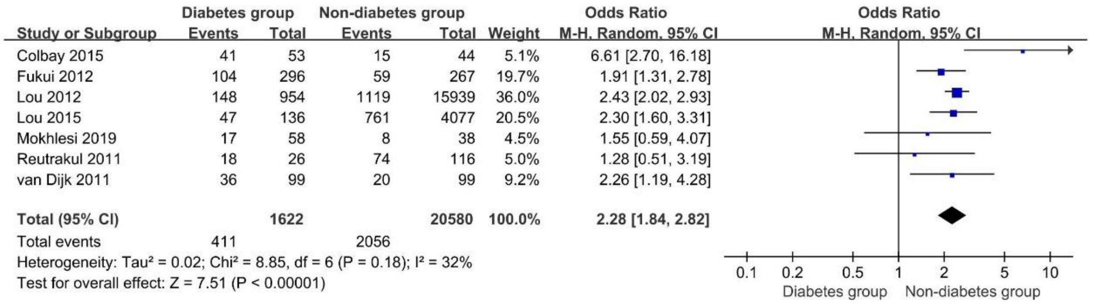

Figure S4. The meta-analysis comparing the occurrence of insomnia between groups with/without diabetes

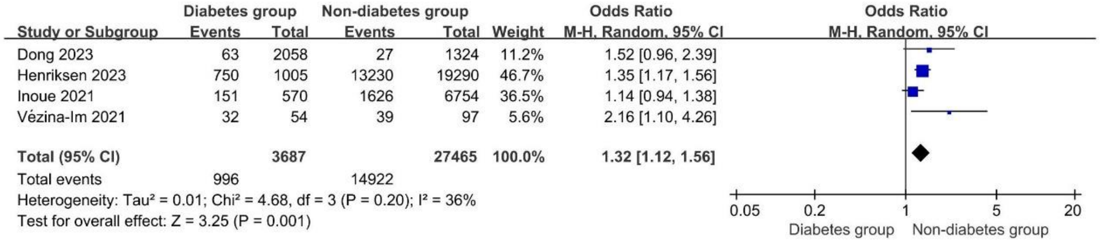

Figure S5. The meta-analysis comparing the glucose levels of groups with high/low sleep quality

Study or Subgroup	Low sleep quality group Mean	SD	Total	High sleep quality group Mean	SD	Total	Weight	Std. Mean Difference IV, Random, 95% CI
Abdu 2023	7.4	2	97	7.7	3	172	5.7%	-0.11 [-0.36, 0.14]
Al-Musharaf 2022	4.7	0.7	70	4.4	0.7	22	2.2%	0.42 [-0.06, 0.91]
Al-Musharaf 2023	92.63	18.65	78	93.9	18.94	409	5.8%	-0.07 [-0.31, 0.18]
Aribas 2015	172	62	31	164	70	47	2.4%	0.12 [-0.34, 0.57]
Bani-Issa 2018	7.96	1.5	90	7.92	1.42	178	5.5%	0.03 [-0.23, 0.28]
Barikani 2019	6.9	3.7	69	6.1	2.1	278	5.3%	0.32 [0.05, 0.58]
Corrado 2024	7.4	1	81	7.3	1	36	3.1%	0.10 [-0.29, 0.49]
Culver 2020	84.5	9.9	9	84	6.6	22	1.0%	0.06 [-0.71, 0.84]
Gozashti 2016	7.9	1.2	87	7.7	1.2	31	2.9%	0.17 [-0.24, 0.58]
Hayashino 2013	7.8	1.3	602	7.8	1.2	911	10.7%	0.00 [-0.10, 0.10]
Hilmisson 2019	81.09	7.21	35	81.09	7.21	37	2.4%	0.00 [-0.46, 0.46]
Huang 2017	7.3	0.5	37	7	0.6	44	2.5%	0.53 [0.09, 0.98]
Martyn-Nemeth 2018	7.2	0.8	22	7.2	1.2	26	1.7%	0.00 [-0.57, 0.57]
Mehrdad 2021	7.84	1.67	185	7.17	1.06	81	5.3%	0.44 [0.18, 0.71]
Meng 2015	8.88	1.95	88	9.03	1.93	244	5.8%	-0.08 [-0.32, 0.17]
Nefs 2015	7.3	1.3	108	7.1	1.3	135	5.5%	0.15 [-0.10, 0.41]
Osonoi 2015	7.1	0.8	77	6.9	1	462	5.9%	0.21 [-0.04, 0.45]
Rizza 2021	5.33	0.32	110	5.23	0.29	163	5.8%	0.33 [0.09, 0.57]
Suteau 2020	7.7	1	156	7.6	0.9	159	6.4%	0.10 [-0.12, 0.33]
Telford 2019	8	1.9	149	7.9	1.7	132	6.1%	0.06 [-0.18, 0.29]
Vargas 2021	102.08	15.58	12	97.35	21.79	14	1.0%	0.24 [-0.54, 1.01]
Yang 2021	7.92	1.63	45	7.35	1.29	25	2.1%	0.37 [-0.12, 0.86]
Zhu 2014	156.77	39.46	97	140.56	40.18	109	5.0%	0.41 [0.13, 0.68]
**Total (95% CI)**			**2335**			**3737**	**100.0%**	**0.14 [0.06, 0.22]**

Heterogeneity: Tau² = 0.01; Chi² = 35.82, df = 22 (P = 0.03); I² = 39%
Test for overall effect: Z = 3.53 (P = 0.0004)

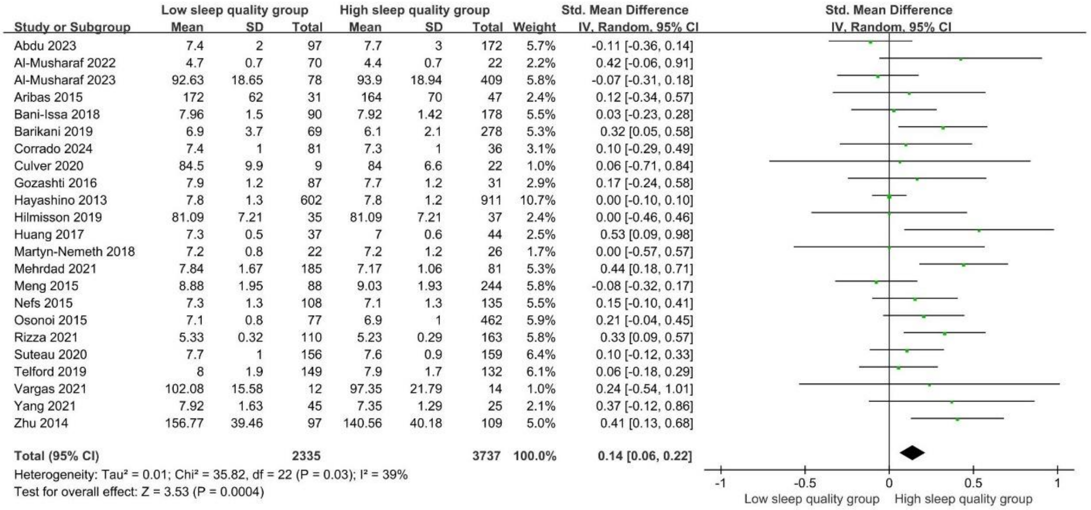

Figure S6. The meta-analysis comparing the glucose levels of groups with/without insomnia

Study or Subgroup	Insomnia group Mean	SD	Total	Non-insomnia group Mean	SD	Total	Weight	Std. Mean Difference IV, Random, 95% CI
Ding 2019	7.72	1.74	359	7.5	1.46	3390	36.9%	0.15 [0.04, 0.26]
Imes 2022	7.9	1.3	108	7.4	1.4	41	5.1%	0.37 [0.01, 0.74]
Jain 2012	7.5	0.5	14	7.6	0.5	5	0.7%	-0.19 [-1.21, 0.83]
O 2023	7.84	1.66	90	7.4	1.29	896	13.0%	0.33 [0.11, 0.55]
Pan 2022	101.39	14.42	60	100.28	14.61	58	5.2%	0.08 [-0.29, 0.44]
Vgontzas 2009	109	32	199	103	38	1022	23.3%	0.16 [0.01, 0.31]
Yoshida 2018	7.1	1	141	7.1	0.8	362	15.7%	0.00 [-0.19, 0.19]
**Total (95% CI)**			**971**			**5774**	**100.0%**	**0.16 [0.07, 0.24]**

Heterogeneity: Tau² = 0.00; Chi² = 7.02, df = 6 (P = 0.32); I² = 15%
Test for overall effect: Z = 3.65 (P = 0.0003)

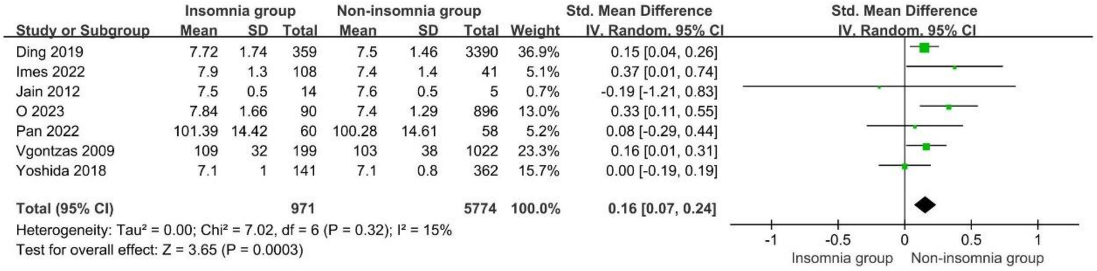

Figure S7. The meta-analysis comparing the sleep quality scores between groups with/without diabetes

Study or Subgroup	Diabetes group Mean	SD	Total	Non-diabetes group Mean	SD	Total	Weight	Std. Mean Difference IV, Random, 95% CI
Adler 2017	39.58	8.74	45	39.6	8.68	45	6.7%	-0.00 [-0.42, 0.41]
Agyekum 2023	7.8	3.9	200	5.5	3	160	7.1%	0.65 [0.44, 0.86]
Bahadur 2022	44.8	6.77	75	43.67	6.07	49	6.8%	0.17 [-0.19, 0.53]
Barone 2015	7.1	1.1	18	7.5	1.6	9	5.6%	-0.30 [-1.11, 0.50]
Caruso 2014	61.3	10.6	16	41.5	14.2	27	5.9%	1.50 [0.79, 2.20]
Gabbs 2022	5.69	2.98	188	5.83	3.28	52	6.9%	-0.05 [-0.35, 0.26]
Haliloglu 2020	6.5	3.4	36	6.6	5	42	6.6%	-0.02 [-0.47, 0.42]
Ishibashi 2020	4.8	0.24	124	3.26	0.22	54	5.7%	6.55 [5.79, 7.31]
Kita 2012	4.4	3.5	74	3.8	3	2788	7.0%	0.20 [-0.03, 0.43]
Mokhlesi 2019	6.4	3.9	258	6.8	3.9	704	7.1%	-0.10 [-0.25, 0.04]
Narisawa 2017	5.7	3.17	622	5.23	2.93	622	7.2%	0.15 [0.04, 0.27]
van Dijk 2011	4.6	0.3	99	4	0.2	99	6.8%	2.34 [1.98, 2.71]
Wu 2023	5.74	2.81	626	3.57	1.71	709	7.2%	0.95 [0.83, 1.06]
Zubair 2018	8.64	3.96	50	4.24	2.72	50	6.7%	1.29 [0.85, 1.72]
Çömlek 2021	3.81	1.91	58	4.72	2.88	40	6.7%	-0.38 [-0.79, 0.02]
**Total (95% CI)**			**2489**			**5450**	**100.0%**	**0.81 [0.40, 1.21]**

Heterogeneity: Tau² = 0.60; Chi² = 582.66, df = 14 (P < 0.00001); I² = 98%
Test for overall effect: Z = 3.89 (P = 0.0001)

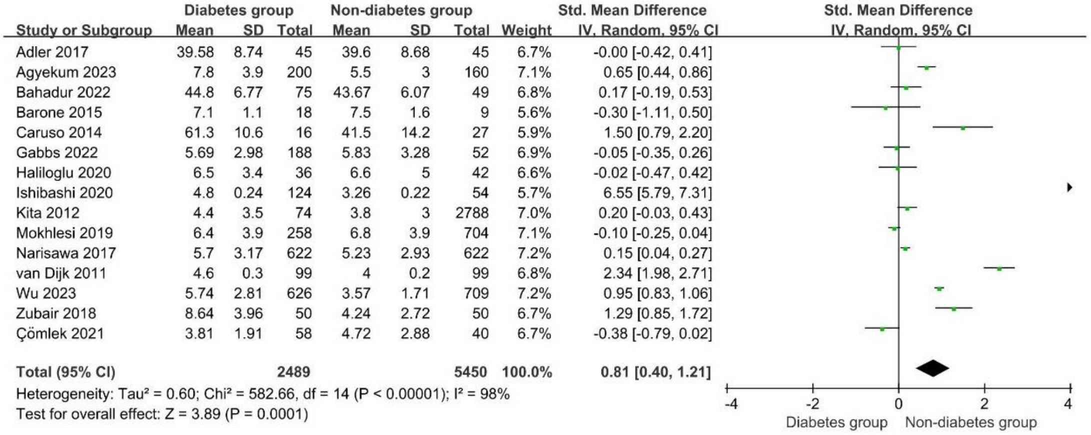

Figure S8. The meta-analysis comparing the sleep quality scores between groups of adverse/normal glycaemic control

Study or Subgroup	Adverse glycemic control Mean	SD	Total	Normal glycemic control Mean	SD	Total	Weight	Mean Difference IV, Random, 95% CI
Cho 2023	5.26	3.53	46	4.7	3.39	50	15.7%	0.56 [-0.83, 1.95]
Hung 2013	6.74	3.23	118	6.07	2.44	1217	84.3%	0.67 [0.07, 1.27]
**Total (95% CI)**			**164**			**1267**	**100.0%**	**0.65 [0.10, 1.20]**

Heterogeneity: Tau² = 0.00; Chi² = 0.02, df = 1 (P = 0.89); I² = 0%
Test for overall effect: Z = 2.33 (P = 0.02)

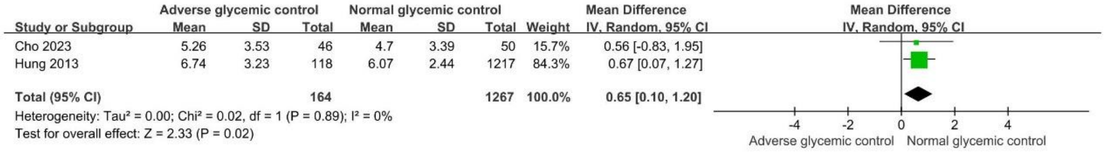

Figure S9. The meta-analysis comparing the sleep efficiency between groups with/without diabetes

Study or Subgroup	Diabetes group Mean	SD	Total	Non-diabetes group Mean	SD	Total	Weight	Mean Difference IV, Random, 95% CI
DePietro 2017	69.8	49.9	73	69.7	22.3	139	1.7%	0.10 [-11.93, 12.13]
Hur 2020	86.25	7.09	30	90.22	4.53	30	21.0%	-3.97 [-6.98, -0.96]
Kostkova 2018	47.8	8.1	44	48.4	0.72	60	29.2%	-0.60 [-3.00, 1.80]
Pillar 2003	87	14	15	92	4	15	4.3%	-5.00 [-12.37, 2.37]
Simon 2018	84.7	3.6	11	86.5	5.2	11	14.8%	-1.80 [-5.54, 1.94]
Trento 2008	83.3	6.5	47	87.6	3.8	23	28.9%	-4.30 [-6.72, -1.88]
**Total (95% CI)**			**220**			**278**	**100.0%**	**-2.73 [-4.31, -1.16]**

Heterogeneity: Tau² = 0.70; Chi² = 6.11, df = 5 (P = 0.30); I² = 18%

Test for overall effect: Z = 3.41 (P = 0.0006)

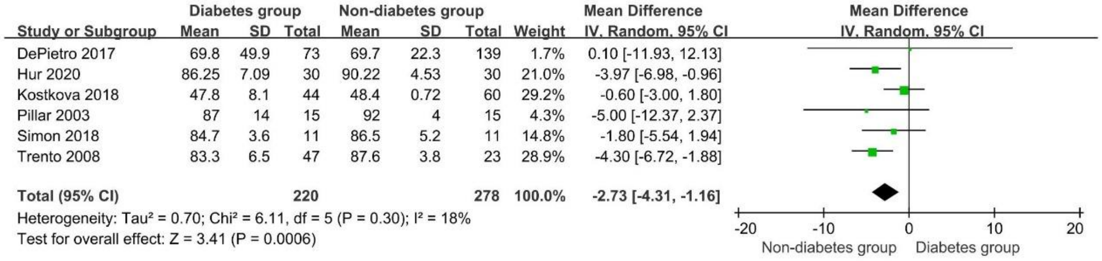

Figure S10. The meta-analysis comparing the risk of diabetes between groups with/without insomnia (related symptoms)

Study or Subgroup	Insomnia (symptoms) group Events	Total	Control group Events	Total	Weight	Risk Ratio M-H, Random, 95% CI
LeBlanc 2018	4564	24146	10062	57087	93.6%	1.07 [1.04, 1.11]
Zou 2022	418	1669	382	1660	6.4%	1.09 [0.96, 1.23]
**Total (95% CI)**		**25815**		**58747**	**100.0%**	**1.07 [1.04, 1.11]**
Total events	4982		10444			

Heterogeneity: Tau² = 0.00; Chi² = 0.05, df = 1 (P = 0.82); I² = 0%

Test for overall effect: Z = 4.54 (P < 0.00001)

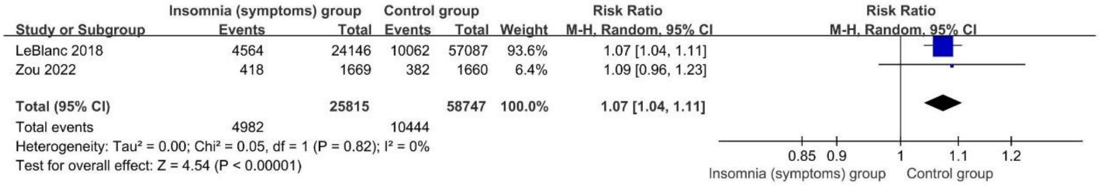

Figure S11. The leave-one-out sensitivity analysis

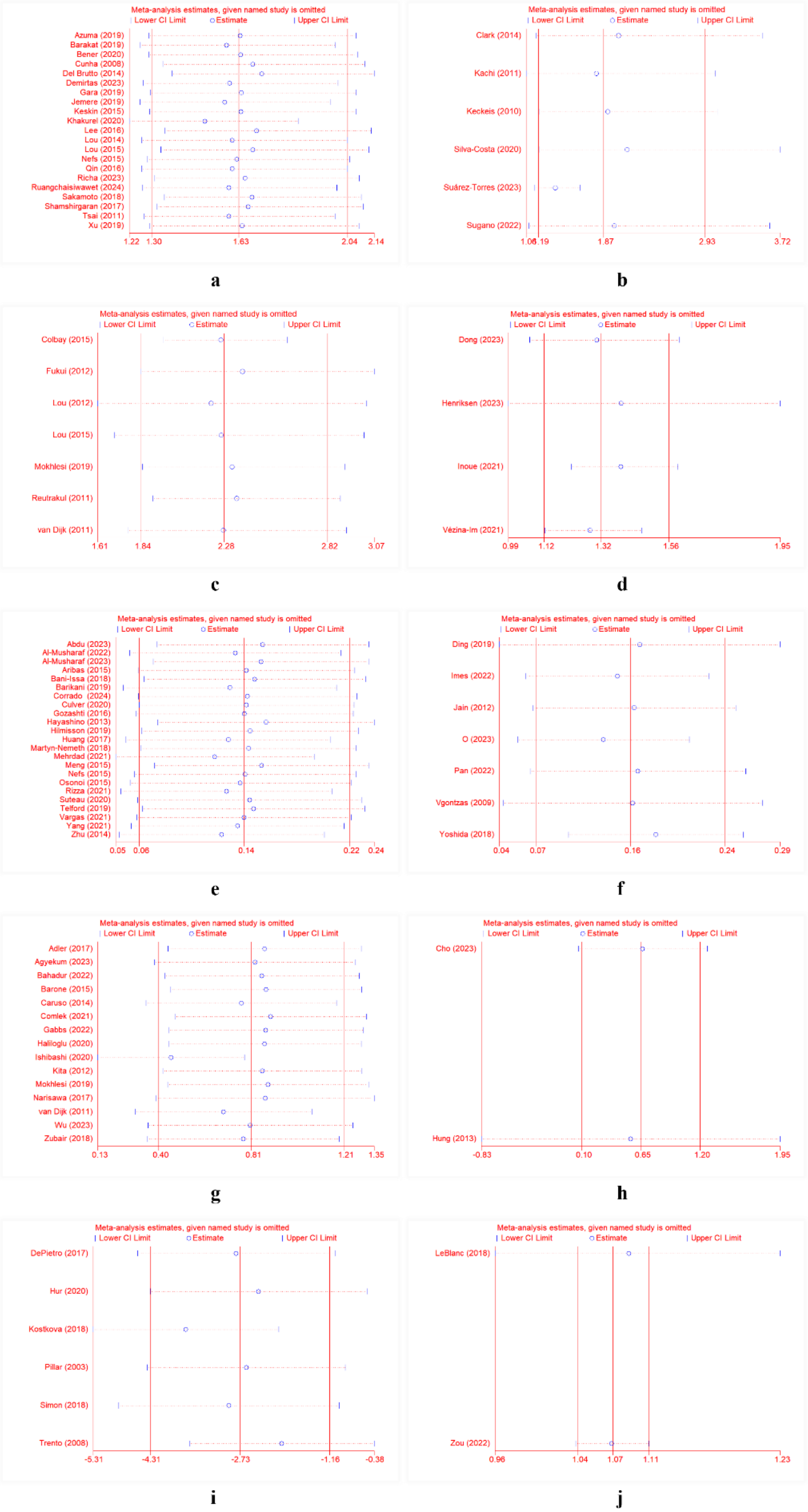

Figure S12. The funnel plots for publication bias

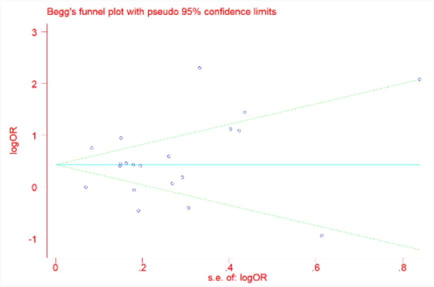

a

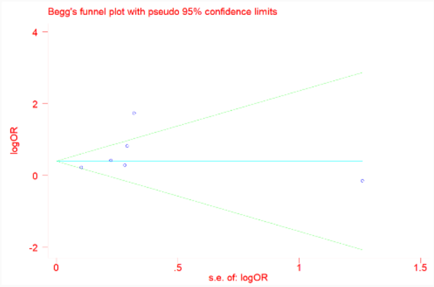

b

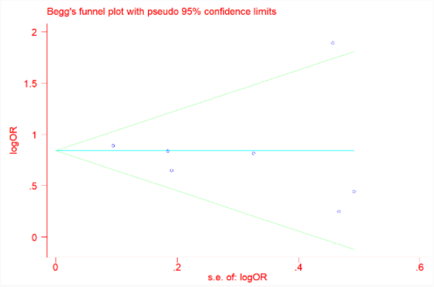

c

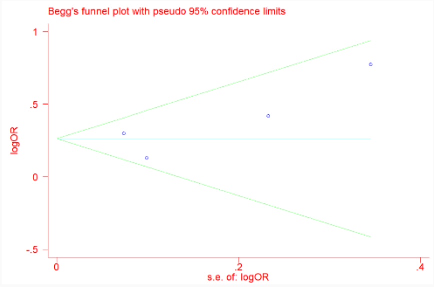

d

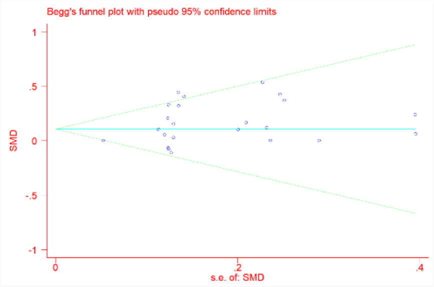

e

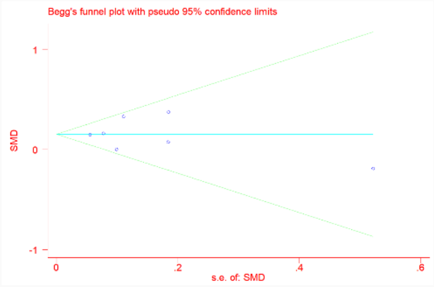

f

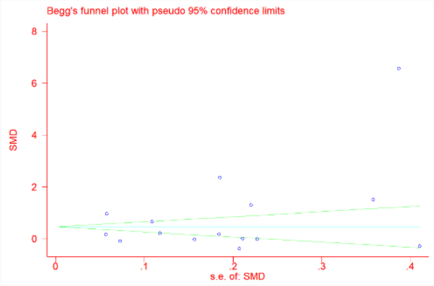

g

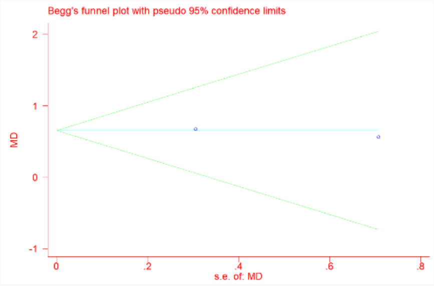

h

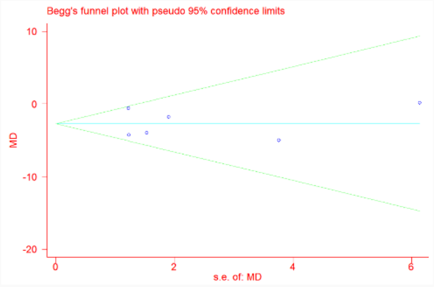

i

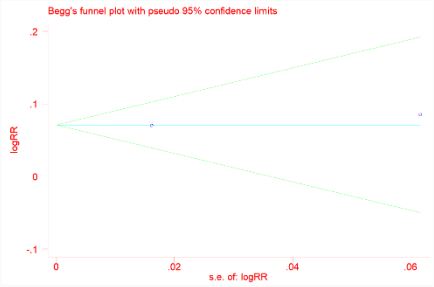

j

Appendix S1

NEWCASTLE - OTTAWA QUALITY ASSESSMENT SCALE

CASE CONTROL STUDIES

Note: A study can be awarded a maximum of one star for each numbered item within the Selection and Exposure categories. A maximum of two stars can be given for Comparability.

Selection

1) Is the case definition adequate?
   a) yes, with independent validation ❈
   b) yes, eg record linkage or based on self reports
   c) no description

2) Representativeness of the cases
   a) consecutive or obviously representative series of cases ❈
   b) potential for selection biases or not stated

3) Selection of Controls
   a) community controls ❈
   b) hospital controls
   c) no description

4) Definition of Controls
   a) no history of disease (endpoint) ❈
   b) no description of source

Comparability

1) Comparability of cases and controls on the basis of the design or analysis
   a) study controls for _________________ (Select the most important factor.) ❈
   b) study controls for any additional factor ❈ (This criteria could be modified to indicate specific control for a second important factor.)

Exposure

1) Assessment of exposure
   a) secure record (eg surgical records) ❈
   b) structured interview where blind to case/control status ❈
   c) interview not blinded to case/control status
   d) written self report or medical record only
   e) no description

2) Same method of ascertainment for cases and controls
   a) yes ❈
   b) no

3) Non-Response rate
   a) same rate for both groups ❈
   b) non respondents described
   c) rate different and no designation

NEWCASTLE - OTTAWA QUALITY ASSESSMENT

SCALE COHORT STUDIES

Note: A study can be awarded a maximum of one star for each numbered item within the Selection and Outcome categories. A maximum of two stars can be given for Comparability

Selection

1) Representativeness of the exposed cohort
   a) truly representative of the average __________________(describe) in the community ❈
   b) somewhat representative of the average _________________in the community ❈
   c) selected group of users eg nurses, volunteers
   d) no description of the derivation of the cohort

2) Selection of the non exposed cohort
   a) drawn from the same community as the exposed cohort ❈
   b) drawn from a different source
   c) no description of the derivation of the non exposed cohort

3) Ascertainment of exposure
   a) secure record (eg surgical records) ❈
   b) structured interview ❈
   c) written self report
   d) no description

4) Demonstration that outcome of interest was not present at start of study
   a) yes ❈
   b) no

Comparability

1) Comparability of cohorts on the basis of the design or analysis
   a) study controls for _______________(select the most important factor) ❈
   b) study controls for any additional factor ❈ (This criteria could be modified to indicate specific control for a second important factor.)

Outcome

1) Assessment of outcome
   a) independent blind assessment ❈
   b) record linkage ❈
   c) self report
   d) no description

2) Was follow-up long enough for outcomes to occur
   a) yes (select an adequate follow up period for outcome of interest) ❈
   b) no

3) Adequacy of follow up of cohorts
   a) complete follow up - all subjects accounted for ❈
   b) subjects lost to follow up unlikely to introduce bias - small number lost - > ________% (select an adequate %) follow up, or description provided of those lost) ❈
   c) follow up rate < _____% (select an adequate %) and no description of those lost
   d) no statement

NEWCASTLE - OTTAWA QUALITY ASSESSMENT SCALE

(adapted for cross-sectional studies)

Selection: (Maximum 3 stars)

1) Representativeness of the sample:
   a) truly representative of the average in the target population (all subjects or random sampling) ❋
   b) somewhat representative of the average in the target population (non-random sampling) ❋
   c) selected group of users
   d) no description of the sampling strategy

2) Non-respondents:
   a) comparability between respondents and non-respondents characteristics is established, and the response rate is satisfactory ❋
   b) the response rate is unsatisfactory, or the comparability between respondents and non-respondents is unsatisfactory
   c) no description of the response rate or the characteristics of the responders and the non-responders

3) Ascertainment of the exposure (risk factor):
   a) validated measurement tool ❋
   b) non-validated measurement tool, but the tool is available or described
   c) no description of the measurement tool

Comparability: (Maximum 2 stars)

1) The subjects in different outcome groups are comparable, based on the study design or analysis. Confounding factors are controlled:
   a) the study controls for the most important factor (select one) ❋
   b) the study control for any additional factor ❋

Outcome: (Maximum 2 stars)

1) Assessment of the outcome:
   a) independent blind assessment ❋
   b) record linkage ❋
   c) self-report
   d) no description

2) Statistical test:
   a) the statistical test used to analyze the data is clearly described and appropriate, and the measurement of the association is presented, including confidence intervals and the probability level (*P* value) ❋
   b) the statistical test is not appropriate, not described or incomplete
